# The bacterial DNA sliding clamp, β-clamp: structure, interactions, dynamics and drug discovery

**DOI:** 10.1007/s00018-024-05252-w

**Published:** 2024-05-30

**Authors:** Signe Simonsen, Caroline K. Søgaard, Johan G. Olsen, Marit Otterlei, Birthe B. Kragelund

**Affiliations:** 1 Linderstrøm-Lang Centre for Protein Science, University of Copenhagen, Ole Maaløes Vej 5, 2200 Copenhagen N, Denmark; 2 Structural Biology and NMR Laboratory, University of Copenhagen, Ole Maaløes Vej 5, 2200 Copenhagen N, Denmark; 3 Department of Clinical and Molecular Medicine, Norwegian University of Science and Technology (NTNU), Trondheim, Norway; 4 Department of Biology, REPIN, University of Copenhagen, Ole Maaløes Vej 5, 2200 Copenhagen N, Denmark

**Keywords:** Clamp loader, PCNA, SLiMs, CBM, APIM, Antibiotic, IDP, PIP-box, PIP-degron, DNA homeostasis

## Abstract

DNA replication is a tightly coordinated event carried out by a multiprotein replication complex. An essential factor in the bacterial replication complex is the ring-shaped DNA sliding clamp, β-clamp, ensuring processive DNA replication and DNA repair through tethering of polymerases and DNA repair proteins to DNA. β -clamp is a hub protein with multiple interaction partners all binding through a conserved clamp binding sequence motif. Due to its central role as a DNA scaffold protein, β-clamp is an interesting target for antimicrobial drugs, yet little effort has been put into understanding the functional interactions of β-clamp. In this review, we scrutinize the β-clamp structure and dynamics, examine how its interactions with a plethora of binding partners are regulated through short linear binding motifs and discuss how contexts play into selection. We describe the dynamic process of clamp loading onto DNA and cover the recent advances in drug development targeting β-clamp. Despite decades of research in β-clamps and recent landmark structural insight, much remains undisclosed fostering an increased focus on this very central protein.

**Supplementary Information:**

The online version contains supplementary material available at 10.1007/s00018-024-05252-w.

## Introduction

**Fig. 1:**
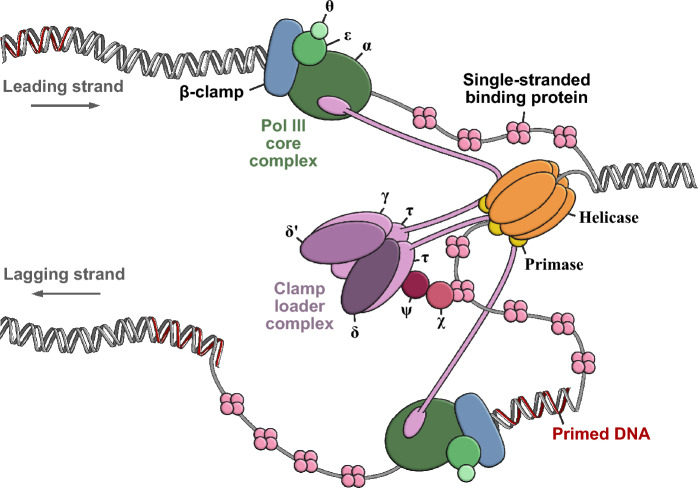
The *E*. *coli* replication complex**.** The clamp loader complex (τ_3_δδ′χΨ—shown in purple) loads β-clamp onto primed DNA, where β-clamp (blue) binds the Pol III core complex (αεθ—shown in green). The clamp loader complex binds the helicase/primase complex (yellow and orange) and can simultaneously bind up to three Pol III core complexes via the C-terminal domains of the three π subunits. χ and ψ links the clamp loader complex to the Single-Stranded Binding (SSB) protein (pink). This figure is inspired by Mulye et al., [16] Lewis et al. [2] and Yao & O’Donnell [3]

DNA replication is a tightly coordinated and complex event. In *E*. *coli*, replication is carried out by a multiprotein replication complex catalysing DNA polymerization at a rate of close to 1000 nucleotides per second [1]. The core replication complex of *Escherichia coli*, consists of twelve proteins divided into three sub-complexes (Fig. 1): (i) a helicase-primase complex, (ii) a polymerase (Pol) III complex, consisting of the Pol III core complex (α, ε, θ subunits) and a DNA sliding clamp, called β-clamp, and (iii) a clamp loader complex comprised of seven subunits; three γ or τ subunits in addition to the δ, δ′, χ and ψ subunits [2, 3]. Other proteins, such as DNA repair and DNA damage avoidance proteins, are linked to or in the proximity of the core complex facilitating an efficient replication process with an error rate of only 5 × 10^−10^ per base pair [1].

The individual proteins and subunits carry out dedicated functions: Helicase is a ring-shaped protein that binds single stranded DNA (ssDNA) and utilizes ATP to unwind double-stranded DNA (dsDNA), while the transiently associated primase produces RNA primers that serve as elongation sites for Pol III. Pol III carries out the simultaneous synthesis of both the leading and lagging DNA strand at the replication fork. The α subunit of the Pol III core complex (αεθ) catalyses the template directed DNA synthesis, while ε is a 3′–5′ proofreading exonuclease removing mismatched nucleotides [2, 3]. The function of θ is to stabilize the ε subunit [4].

Efficient DNA synthesis of the Pol III core complex depends on β-clamp, which acts as a processivity factor in the Pol III complex. β-clamp is a homodimeric, ring-shaped protein that encircles dsDNA [5], attaches the Pol III core complex to the primed site and then slides along DNA during replication, while tethering the polymerase to DNA and ensuring efficient DNA synthesis [2, 3]. β-clamp also interacts with other proteins, including polymerases involved in translesion synthesis (TLS) [6–8] and DNA mismatch repair [9, 10]. It therefore serves as a key hub protein important for DNA homeostasis.

β-clamp is loaded onto DNA by the clamp loader complex (τ_n_γ_(3−n)_ δδ′χψ) in an ATP-dependent mechanism. τ is a full-length protein, where γ is its truncated version produced by a ribosomal frameshift event during translation of the *dnaX* gene [11]. Only the so-called γ complex τ_n_γ_(3−n)_ δδ′ pentameric subunit is required to bind and open the β-clamp dimer interface prior to DNA loading [2, 3, 12]. The clamp loader also functions to unload β-clamp from DNA for clamp recycling [13, 14]. The ψ and χ accessory subunits of the clamp loader have other functions; φ binds to χ and to the γ/τ subunit of the clamp loader, while χ interacts with the single-strand binding (SSB) protein that coats ssDNA, thereby tethering the clamp loader complex to the lagging strand during synthesis [2, 3, 12]. The clamp loader complex further interacts with helicase and the Pol III core complex, with τ of the clamp loader mediating the interactions with both helicase and Pol III α. Given that one clamp loader complex can contain up to three τ subunits, it can bind up to three Pol III core complexes simultaneously, however, the most dominant clamp loader composition contains one γ and two τ subunits [15]. The clamp loader therefore also serves a central role in organizing the replisome, connecting polymerases at the lagging- and leading strands with the helicase (Fig. 1) [2, 3].

DNA sliding clamps are conserved across all domains of life [17]. Higher eukaryotes have two β-clamp orthologues, the proliferating cell nuclear antigen (PCNA) [18] and the 9-1-1 complex [19]. The T4 bacteriophage also has a DNA sliding clamp [20]. Although β-clamp is a dimer, and PCNA, 9-1-1 and the T4 sliding clamps exist as trimers, their overall 3D structures are conserved despite the divergence that occurred some three billion years ago. Despite completely different sequences (11% sequence identity between human PCNA and *E*. *coli* β-clamp domains II and III), the structures of various sliding clamps align with an RMSD less than 3.5 Å [21]. Both β-clamp and PCNA are known to interact through short linear sequence motifs (SLiMs) in their interaction partners. SLiMs are often located in intrinsically disordered regions (IDR) [22], and this is also the case for SLiMs interacting with PCNA [23] and to some extend for β-clamp (Fig. S1). β-clamp binding partners interact via a SLiM called the clamp binding motif (CBM), defined as Qφx[L/M][F/L] and Qφx[L/M]x[F/L] (φ = aliphatic, x = any residue), as discussed below. PCNA binding partners interact via the PCNA interacting protein (PIP) box QxxφxxΩΩ (Ω = aromatic) [23, 24], the PIP degron QxxφTDΩΩxxx(R/K) [25] or the AlkB homologue 2 PCNA-interacting (APIM) motif [K/R]-[F/Y/W]-[L/I/V/A]-[L/I/V/A]-[K/R] [26].

DNA sliding clamps are considered interesting drug targets. PCNA is a target for anti-cancer drugs [27–30] and β-clamp a target for antimicrobial drugs (reviewed in [27]). β-clamp is important in TLS, and because TLS-introduced mutations are involved in the development of resistance to antibiotics, targeting β-clamp could be a way of combating antimicrobial resistance [31]. Although β-clamps are promising therapeutic targets, very little focus on understanding the molecular basis for their function as antibacterial drug targets has been made. This knowledge gap hampers directed targeting in disease control and more active research into these exciting hub proteins is needed to push this field forward. In this review we exclusively focus on the *E*. *coli* β-clamp with emphasis on its structure, interactions, and dynamics and, given its relevance in antimicrobial resistance, on drug discovery in targeting β-clamp.

## β-clamp structure

**Fig. 2:**
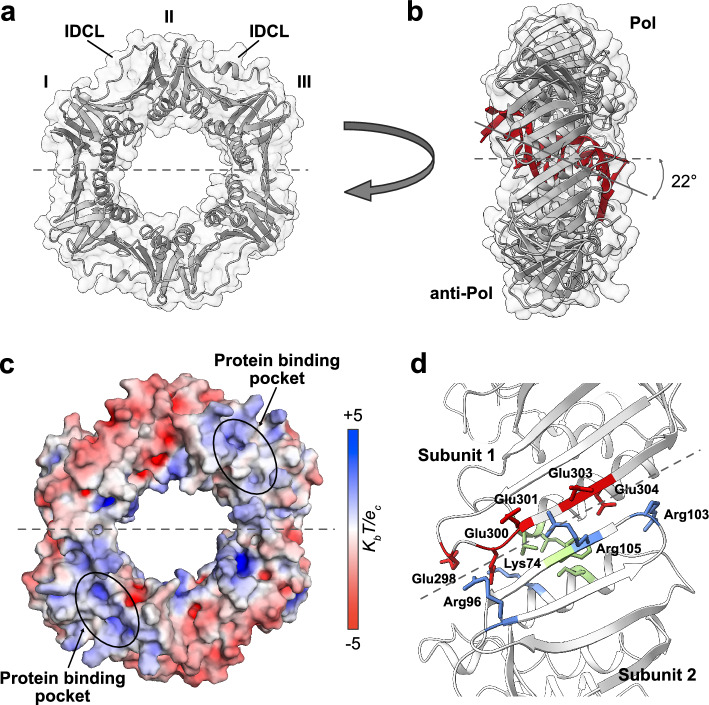
Structure of β-clamp. Dashed lines indicate the border between the two subunits. **a** Crystal structure of *E. coli* β-clamp (PDB ID: 2POL [32]). I II and III indicate the three domains. IDCL: interdomain connecting loops. **b** Crystal structure of β-clamp bound to primed DNA shown in red (PDB ID: 3BEP [5]) with the polymerase binding (Pol) side and the other (anti-Pol) side indicated. **c** Electrostatic surface potential computed by the APBS Electrostatics plugin in PyMOL (PDB ID: 1MMI [33]). Red areas indicate an overall negative charge and blue areas overall positive charge. **d** Close-up on the β-clamp dimer interface (PDB ID: 2POL [32]). Key interacting residues at the interface are shown as sticks and named. Negatively charged residues are coloured red, positively charged residues blue and the hydrophobic residues Ile78, Phe106, Ile272 and Leu273 are shown in green

The first crystal structure of β-clamp was published in 1992 by Kong et al*.* (Fig. 2a) [32]. β-clamp has the shape of a starlike toroidal ring and forms a head-to-tail homodimer with the C-terminal region of one subunit interacting with the N-terminal region of the other. Each β-clamp subunit is composed of three structural domains (I, II and III) linked by so-called interdomain connecting loops (IDCLs). The domains share topology, each composed of two four-stranded antiparallel β-sheets and two α-helices, totalling 24 β-strands and twelve α-helices. Even though the domains have similar 3D structures (RMSDs of C^α^-atoms are 1.85 Å (I and II) 1.77 Å (I and III), and 2.03 Å (II and III) excluding the IDCLs), pairwise sequence alignments show only 9–16% sequence identity [32].

The twelve α-helices of the β-clamp dimer form the inner ring of the toroidal structure and the β-sheets the outer surface. The diameter of the central channel is ~35 Å [32], large enough to accommodate DNA. The channel includes many water molecules, and transient interactions between β-clamp, water and DNA are proposed to be important for mediating the sliding action as β-clamp moves along DNA [33]. This water-mediated sliding mechanism has been reviewed in [34]. Only a single crystal structure of β-clamp with DNA is available. Here, the DNA inside the channel is tilted by 22° (Fig. 2b). However, in a cryo-EM structure of the complex between β-clamp, Pol III α, exonuclease, the C-terminal domain of τ and DNA, the DNA is less tilted and located in an almost perpendicular orientation relative to β-clamp [35]. The diffusion constant for β-clamp sliding along DNA is remarkedly slower than its predicted diffusion constant through water alone, which may imply that the attractive forces between β-clamp and DNA slow down DNA sliding [36]. Although little is known about the DNA sliding mechanism, molecular dynamics simulations suggests that the speed of DNA sliding clamps along DNA is mainly determined by the shape and geometry of the inner channel and to a smaller extend by the electrostatic potential in the channel. The elliptical shape of the β-clamp channel slows down diffusion compared to PCNA which has a more circular channel [37].

Not surprisingly, the surface of the channel is strongly positively charged providing favourable interactions with the negatively charged phosphate groups of the DNA backbone (Fig. 2c). Some of the basic side chains lining the inner channel are highly flexible but become ordered upon DNA interaction [5, 33]. The outer surface of β-clamp has two sides, the side for Pol III binding, which we term the Pol side, and the opposite side here termed the anti-Pol side (Fig. 2b). Both sides are mainly negatively charged except for the areas around the partner protein binding pockets (Fig. 2c).

### The dimer interface is stabilized by hydrophobic and electrostatic interactions

The β-clamp dimer is stable even at nanomolar concentrations with an affinity (*K*_D_) estimated to be 6.5–60 picomolar [38, 39] and a half-life of ~43 h [39]. However, when bound to DNA, and in the absence of the clamp loader, the half-life for spontaneous DNA dissociation ranges between ~1 and 2 h at 37 °C [13, 38, 40]. If not caused by condition variations, this difference could indicate that β-clamp is destabilized when bound to DNA [39]. The dimer is held together by at least four hydrogen bonds formed between the β-strands from different subunits, resulting in a continuous β-sheet across the subunits. The dimer is further stabilized by hydrophobic and electrostatic interactions. In the core of the interface, Ile78 and Phe106 from one subunit pack against Ile272 and Leu273 of the other forming a small hydrophobic core (Fig. 2d) [32]. The importance of this core is reflected both in the double mutant Ile272Ala;Leu273Ala, which results in a weakened β-clamp dimer [41, 42], as well as the Ile272Ala;Leu82Glu double mutant, which exhibits reduced thermostability and weakened dimerization [43]. Surrounding these, five negatively charged residues from one subunit form six intermolecular ionic interactions with four positively charged residues of the other (Fig. 2d). These ion pairs form between Lys74 and Glu298, Lys74 and Glu300, Arg96 and Glu300, Arg103 and Glu304, Arg105 and Glu301 and Arg105 and Glu303, respectively. The ion pairs between Arg96-Glu300 and Arg103-Glu304 involve charged groups that are solvent inaccessible, thus forming very strong ionic interactions [32] and mutating Arg103 weakens the dimer resulting in faster dimer dissociation and reduced thermostability [44]. The stability of the β-clamp dimer is therefore highly dependent on the ionic strength, and complete dissociation of the dimer at 0.5–1 M NaCl is reported [39, 44].

### β-clamp conservation

#### β-clamps are structurally conserved

To investigate the structure and sequence conservation among bacterial sliding clamps, we identified eight β-clamp structures from different bacterial organisms. Selection was based on the criteria of i) absence of any bound ligands (except for ions), ii) crystal structures with a resolution better than 3 Å, iii) representing wild type proteins, and iv) structural details are published. Furthermore, they were selected to represent both Gram-negative (5 structures) and Gram-positive (3 structures) bacteria. The Pairwise Structural Alignment tool from RCSB (https://www.rcsb.org/alignment) was used to align the eight structures employing the TM-align algorithm [45]. Chain A of all structures were aligned to Chain A of the *E*. *coli* β -clamp (1MMI) [33], which served as reference.

**Fig. 3:**
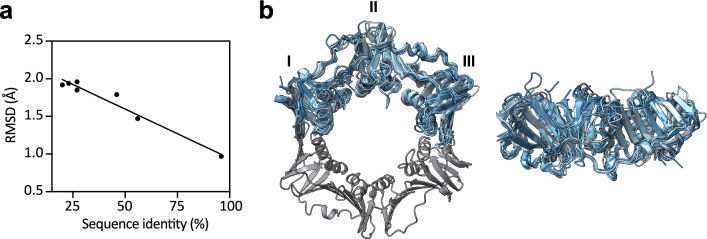
Structural conservation among β-clamps from different bacterial organisms. **a** RMSDs of the different β-clamp structures relative to the reference structure from *E. coli* plotted against their sequence identity with *E*. *coli* β-clamp. **b** Overlay of the β-clamp structures listed in Table S1, with the *E*. *coli* reference structure coloured grey and the other structures shown in blue ranging from high sequence identity (dark blue) to low sequence identity (light blue)

In general, the structures of β-clamps from Gram-negative organisms share the highest sequence identity with β-clamp from *E*. *coli* except for *Helicobacter* *pylori,* which only shares 20% sequence identity with the reference structure (Table S1). While the sequence identity correlates with the RMSD of the structures relative to the reference (Fig. 3a), the overall RMSDs do not exceed 2 Å, even for the most distal orthologs. Thus, there is an impressively high structural conservation of the β-clamps. The aligned structures are depicted in Fig. 3b, with highest structural similarity in the folded part of the structures and more variation within the loops.

#### β-clamps are conserved in their binding interface

**Fig. 4:**
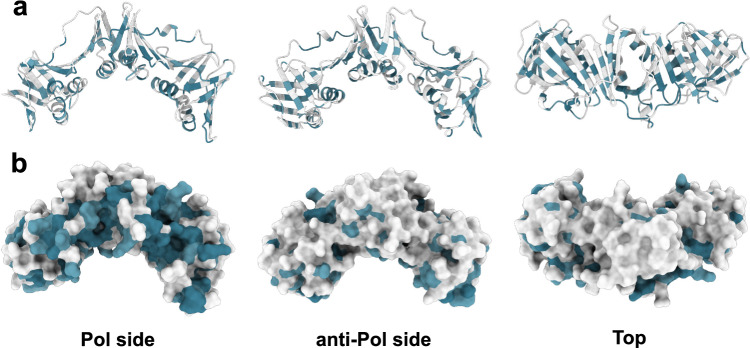
Structure conservation shown on the structure of *E*. *coli* β-clamp. Colouring is according to the structural sequence alignment in Fig. S1. PDB ID: 1MMI (only one subunit is shown). Conserved residues are coloured blue. **a**: Cartoon representation. **b**: Surface representation

Based on the structural alignment we extracted the corresponding sequence alignment using the TM-align algorithm (Fig. S2). Thus, residues are aligned based on their positions in the 3D structures; consequently, residues that are not visible in the structures, e.g., flexible regions, are not represented in the alignment. From this alignment, clear clusters of conservation are observed in all three domains of β-clamp (Fig. 4) located in both structured parts and in loops. Notably, there is a pattern in several of the β-strands where every second residue is conserved (Fig. 4a), and the side chains of these residues mostly point towards the core of the protein. Oppositely, most of the solvent exposed residues are not conserved, except for the positively charged residues lining the inner channel and the residues on the Pol side of β-clamp, known to be involved in protein interactions (Fig. 4b). Furthermore, Ile78, Phe106, Ile272 and Leu273 involved in stabilizing the dimer interface [32, 41], are all conserved. Of the charged residues in the dimer interface, only Arg103, Glu298 and Glu304 are more than 70% conserved, indicating that electrostatic interactions here are not as conserved. Thus, it seems convincing that the evolutionary restrictions on β-clamp have been to conserve the 3D structure in addition to its interactions with DNA and protein partners, highlighting the functional conservation.

### β-clamp interactions

Β-clamp interacts with a large variety of proteins involved in DNA replication and repair including replicative DNA- and TLS polymerases. Known β-clamp binding partners and their peptide counterparts are listed in Table 1**,** including affinities and validation methods. Generally, affinities for peptide ligands are weak and in the micromolar range (from ~1 to 210 µM), whereas full-length proteins typically bind in the low micromolar/high nanomolar range, similar to the affinity for DNA (120–453 nM). The ligands with the highest affinities (low nanomolar) are the clamp loader γ complex and the full-length Pol III core complex when β-clamp is bound to DNA.

**Table 1:** β-clamp interaction partners and quantified affinities

Partner	Function	Affinities^a^	Validation method(s)
Duplex DNA [5]		*K*_D_ = 453 nM [5]	Fluorescence [5]
Primed DNA [5]		*K*_D_ = 120 nM [5]	Fluorescence [5] X-ray crystallography (PDB ID: 3BEP) [5]
Pol I [10]	DNA replication and repair	ND	Native PAGE [10]
Pol II [6, 46, 47]	DNA repair	CBM peptides:	
		TLMTGQLGLF: *K*_D_ = 1.7 µM (data not shown) [46]	ITC (data not shown) [46]
		ILPFIEDNFATLMTGQLGLF: *K*_D_ = 3.5 µM [47]	Fluorescence [47]
		Full-length Pol II: *K*_D_ = 120 nM [47], *K*_D_ = 31.6 nM [6]	SPR [6]
			X-ray crystallography with peptide (PDB ID: 3D1E) [46]
Pol III α [14, 35, 46–54]	DNA replication	C-terminal CBM peptides:	
		SEQVELEFD: *K*_D_ = 1.42 µM [46]	ITC [46]
		RLLNDLRGLIGSEQVELEFD: *K*_D_ ~3 µM [48], *K*_D_ = 3.06 µM [46], *K*_D_ = 3.2 µM [47]	Fluorescence [47, 48], ITC [46]
		GATWRVSPSDRLLNDLRGLIGSEQVELEFD: *K*_D_ = 3.95 µM [46]	ITC [46]
		Internal CBM peptides:	
		IGQADMFGV: *K*_D_ ≥ 15 µM [46], *K*_D_ = 2.7 µM [49]	ITC [46], SPR [49]
		Full-length α: *K*_D_ = 108 nM [53], *K*_D_ = 0.11 µM [47], *K*_D_ = 0.2–1µM [52], *K*_D_ = 0.8 µM [51], *K*_D_ = 1.5 µM [50], *K*_D_ = 1.2 µM [54]	SPR [51, 53], fluorescence [47], SEC [52, 54], ITC [50]
		Full-length α + ε: *K*_D_ = 0.3 µM [54]	SEC [54]
		Pol III core (α + ε + θ): *K*_D_ ~50 nM [48], *K*_*D*_ = 51 nM [47]	Fluorescence [47, 48]
		Pol III core with β-clamp bound to DNA: *K*_D_ < 5 nM [14]	SEC [14]
			X-ray crystallography with C-terminal peptide (PDB ID: 3D1F) [46] Cryo-EM of β-clamp:α:ε complex (mutated α and ε variants - PDB ID: 5FKV) [35]
Pol III ε [35, 54, 55]	Proofreading 3′–5"exonuclease	GGQTSMAFAV: *K*_D_ ~210 µM [55]	SPR [55]
			Chemical crosslinking MS [54]
			Cryo-EM of β-clamp:α:ε complex (mutated α and ε variants - PDB ID: 5FKV) [35]
Pol IV [6, 7, 56, 57]	Translesion synthesis (TLS)	CBM peptides:	
		RQLVLGL: *K*_D_ = 1.6 µM [57]	ITC [57]
		VTLLDPQMERQLVLGL: *K*_D_ estimated to be in the µM range. *K*_D_ ~1.2 µM if inhibitions require two occupied binding sites and ~8 µM if it only requires one occupied binding site [56]	Competition replication experiment [56]
		Full-length Pol IV: *K*_D_ = 465 nM [6]	SPR [6]
			X-ray crystallography with peptide (PDB ID: 1OK7) [56] and little finger (LF) domain (PDB ID:1UNN) [7]
UmuC (Pol V) [8]	Translesion synthesis (TLS)	ND	X-ray crystallography with peptide (PDB ID: 4K74) [8]
UmuD (Pol V) [58–60]	Translesion synthesis (TLS)	Fulll length UmuD: *K*_D_ = 5.5 µM [58]	Fluorescence [58]
			Affinity chromatography [59]
			Chemical crosslinking [60]
δ subunit (clamp loader complex) [41, 53, 61, 62]	Clamp loading onto DNA	Full length δ and monomeric β-clamp Ile272Ala;Leu273Ala: *K*_D_ ~7.5 nM [41]	SPR [41]
		Full length δ and dimeric β-clamp: *K*_D_ ~460 nM [41], *K*_D_ ~7–10 nM [62], *K*_D_ = 17.5 nM and 160 nM (two δ binding events per β-clamp dimer with negative cooperativity) [53]	SPR [41, 53, 62]
			X-ray crystallography with full-length δ (PDB ID: 1JQJ) and shorter variant (1–140—PDB ID: 1JQL) [61]
τ complex [5, 6, 62–65]	Clamp loading onto DNA + organizing Pol III complex	Without ATP: *K*_D_ = 2.8 µM (γ_3_δδ′χψ) [62]	SEC [62]
		With ATP: *K*_D_ = 3.2 nM [62] (γ_3_δδ′χψ), *K*_D_ = 1.6 nM (Supplementary data) [5], *K*_D_ = 2.7 nM [63] (γ_3_δδ′χψ), 3.6 nM (τ_3_δδ′χψ) [63], *K*_D_ = 27 nM (γ_3_δδ′χψ) [6], 0.9 nM (γ_3_δδ′) [65], 0.4 nM (γ_3_δδ′ψ) [65], 0.7 nM (γ_3_δδ′χψ)[65]	SPR [6, 62, 63], Fluorescence anisotropy [5, 65]
			Cryo-EM of β-clamp:γ complex with and without DNA [63, 64]
MutL [9]	Mismatch repair	ND	X-ray crystallography with the regulatory domain of MutL (PDB ID: 6E8E) [9]
MutS [10]	Mismatch repair	ND	Native PAGE [10]
Hda [49, 66–68]	Regulation of replication initiation	PAQLSLPLYL: *K*_D_ = 0.38 µM [49]	SPR [49]
			Plate binding assay [66]
			Pull-down assay [66, 68]
			X-ray crystallography with full-length Hda (PDB ID: 5X06) [67]
DNA ligase [10]	Joins nascent DNA fragments	ND	Native PAGE [10]

^a^Note that differences in *K*_D_*s* might be caused by experimental condition variations such as buffer components, salt concentration, temperature, and experimental method

*ND* not determined, *SPR* surface plasmon resonance, *SEC* size exclusion chromatography, *ITC* isothermal titration calorimetry

#### Dissection of the consensus binding motif of β-clamp interaction partners

**Fig. 5:**
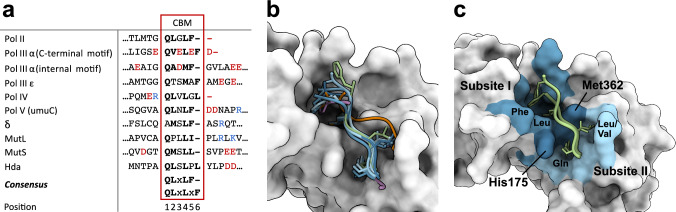
The CBMs and the β-clamp binding pocket.** a** Alignment of CBMs of β-clamp ligands. Positively charged residues are coloured blue and negatively charged residues are red. The C-terminus of C-terminal CBMs is indicated by a red hyphen. Bold residues indicate key residues of the CBMs. **b** Structure of the 5/6 residue CBMs from peptide ligands and full-length protein/domains. The structure of β-clamp (in complex with Pol III peptide) (PDB ID: 3D1F [46]) is shown as grey surface, while ligands of aligned structures are shown in cartoon representation with important residues highlighted as sticks. CBMs from Hda (PDB ID: 5X06 [67]), Pol IV LF domain (PDB ID: 1UNN [7]), Pol II peptide (PDB ID: 3D1E [46]), Pol III peptide (PDB ID: 3D1F [46]) and Pol IV peptide (PDB ID: 1OK7 [56]) are shown in variations of blue, while CBMs of the clamp loader δ subunit 1-140 (PDB ID: 1JQL [61]), Pol V peptide (PDB ID: 4K74 [8]) and MutL (PDB ID: 6E8E [9]) are shown in purple, green and orange, respectively. **c**: The peptide binding pocket of β-clamp. Residues of subsite I are shown in blue, residues of subsite II in light blue and H175 and M362 are shown in dark blue. The pentameric CBM of Pol II peptide (PDB ID: 3D1E [46]) is shown in dark green and the hexameric CBM of Pol III peptide (PDB ID: 3D1F [46]) in light green. Important residues of these binding motifs are shown as sticks and highlighted

All β-clamp binding partners interact via SLiMs [69–71], known as CBMs (Fig. 5a). Some CBMs reside in the C-terminal tail of the ligands (Pol II, Pol IV, and the C-terminal motif of Pol III α), the CBM of Hda is located in the disordered N-terminus, whereas other CBMs are located internally in loops of otherwise folded parts of the partners (Fig. S1). A consensus motif for CBMs (QL(S/D)LF) was identified in 2001 by Dalrymple et al. [72] and has since been widely used to search for new β-clamp binders. However, this motif cannot explain some of the variations seen in the CBMs. In this section, we scrutinize the CBMs of known β-clamp ligands and compare them to mutational studies.

Two types of CBMs have been observed in β-clamp binding partners—a pentameric and a hexameric motif. The first residue (position 1) of the CBMs is a conserved Gln, followed by a (large) aliphatic amino acid. Two other important residues are Leu or Met at position 4 followed by a hydrophobic amino acid, preferably Phe or Leu at position 5 or 6 (Fig. 5a). Using molecular modelling approaches, Wolff et al. [57] found that the Gln and three Leu residues of the Pol IV CBM (RQLVLGL) primarily contribute to the interaction. Gly and Val do not interact with β-clamp, hinting that the conserved residues are the primary interacting residues.

The Gln at the first position contributes significantly to β-clamp binding; mutating Gln to Ala in the internal CBM of full-length Pol III α (QADMF) increased *K*_D_ from 0.8 to 4.2 µM, resulting in reduced activity [51], in accordance with the same mutation in full-length Hda impairing β-clamp binding [68]. Replacing Gln in the consensus peptide QLDLF with Glu, Ala or Gly greatly reduced the affinity, whereas substituting Gln with Asn showed only modest effects [73]. Altering Gln of the Pol V CBM (QLNLF) resulted in weaker binding, but not a complete abrogation of binding [74]. To our knowledge, the only known example of a natural β-clamp ligand that lacks Gln in the first position is the δ subunit. Although the δ subunit binds β-clamp with nanomolar affinity (Table 1), peptides containing the δ CBM (AMSLF) are poor competitors of the δ:β-clamp and Pol III α:β-clamp interactions [49] suggesting the motif itself to have low affinity. Altogether, these results suggest that the conserved Gln is important to increase the affinity to β-clamp, yet this residue is not essential since some level of interaction persist even in its absence. Since the consensus motif identified by Dalrymple et al. in 2001 [72] contains a Gln at position 1, the search for potential interaction partners might be biased towards Gln-containing motifs overlooking others, as previously suggested for PCNA binding motifs [23]. Moreover, detection of low affinity binders can be experimentally challenging, possibly contributing to the absence of CBMs lacking Gln.

The second residue of the CBM is most often an aliphatic amino acid but there are exceptions (Fig. 5a). Peptide CBMs from different binding partners generally interact with β-clamp with low micromolar affinity (Table 1), with the exception of the CBM of the Pol III ε subunit (QTSMAF), binding much weaker (*K*_D_ of ~210 µM) [55]. This motif also diverges from the canonical CBM in the second position. Although the internal motif of Pol III α (QADMF) binds with an affinity of 2.7 µM [49], others have reported weaker binding with affinities of ≥ 15 µM [46]. Interestingly, replacing Ala of the internal Pol III α CBM to Leu increases affinity [49], and replacing Leu at the second position of an Ac-QLDLF consensus motif with Ala, decreases the peptide affinity ~40fold [73], hinting that Ala is too small to make favourable interactions with β-clamp. Although substitution of Leu at position 2 in the QLDLF consensus motif with Val, Ala or Gly decreases the affinity, these mutations were not as detrimental as mutations of the Gln [73]. Therefore, position 2 of the CBM appear tolerant to substitutions, with Leu being the more favourable.

As evident from Fig. 5a, the third position of the CBM varies greatly. Mutations at this position are largely tolerated, although a slight preference towards Asp exist [49, 73]. Interestingly, mutation of the otherwise non-conserved third position of the CBM of the internal Pol III α motif from Asp to Ala, Val or Ser severely impairs binding to β-clamp [72]. This could indicate that negatively charged Asp of the internal Pol III α CBM might compensate for the less favourable Ala at position two and Met at position four. The fourth residue of the CBM is a key interacting residue of the CBMs [47, 68, 74, 75]. Only Leu and Met occupy this position, with Leu being the more abundant (Fig. 5a). The internal motifs of Pol III α and Pol III ε are the only CBMs containing Met at the fourth position. These CBMs have lower affinities compared to other peptide motifs (Table 1), suggesting that Met causes the lower affinities, although the second position differs as well. In support of this observation, Wijffels et al. [75] found that substitution of Met to Leu in the internal motif of Pol III α improved the inhibitory activity, whereas peptides with Ile, Phe and Trp at the same position showed weaker inhibition.

Position 5/6 of the CBM is acknowledged to be important for β-clamp binding [47, 74]. Mutating Phe of the internal motif of Pol III α (QADMF) in both the full-length protein [51] and the CBM alone [49], reduce binding and completely abolish Pol III activity [51]. Although both Phe and Leu (and Ile) are tolerated at this position (Fig. 5a), Phe has slightly improved affinity over Leu [57, 73]. Substituting Phe with Ala or Gly in the consensus motif (QLDLF) or with Trp in the internal CBM of Pol III α significantly weakens binding to β-clamp [73, 75], implicating that this position has strong requirements to the type of amino acid occupying this position. Whether the CBM is pentameric or hexameric appear not to impact affinity (Table 1). Whereas removing Gly in the Pol IV peptide sequence (RQLVLGL) impair the interaction by 2–3 fold, replacing the terminal LGL with LF has no effect [57]. Several studies have identified the pentameric QLDLF consensus motif to be the strongest peptide binding sequence [49, 51, 73], still, a phage display experiment found the LQLELDF peptide containing a hexameric motif to be the most abundant, binding with low µM affinity [46].

In conclusion, the conserved Gln is an important contributor to the affinity of the CBM, but not necessary for binding. The second position is not as restricted but most often occupied by an aliphatic amino acid, with Leu being the most favourable. While the third residue is less important, the fourth and fifth/sixth positions of the CBM are highly important and more restricted in the type of amino acids allowed. The fourth position can be occupied by Leu and Met, and the fifth/sixth position can contain both Phe, Leu and Ile, where the Leu/Phe combination of the last two hydrophobic residues is the most favourable. Hence, the canonical CBMs found in natural β-clamp ligands identified so far can be defined as Qφx[L/M][F/L] and Qφx[L/M]x[F/L], where φ is an aliphatic residue and χ any residue, whilst the CBMs exhibiting the highest affinities follow the consensus sequence QLxLF, close to that identified by Dalrymple et al. [72], and QLxLxF.

#### The CBMs of β-clamp ligands bind the same binding pocket

So far, the CBMs of all known partners bind to the same binding pocket of β-clamp (Fig. 5b). The pocket has two distinct subsites (Fig. 5c); subsite I comprised of β-clamp residues Arg152, Leu155, Thr172, Gly174, His175, Arg176, Leu177, Pro242, Arg246, Val247, Val360 and Met362, and subsite II constituted by residues His175, Asn320, Tyr323, Val344, Met362, Pro363, Met364 and Arg365. Fig. S3 shows the contacts formed between β-clamp and various peptides and full-length protein ligands or protein domains whose complex has been solved using X-ray crystallography.

Residues His175 and Met362 bridge the two subsites (Fig. 5c). In the free state, Met362 can be oriented towards His175 forming a cleft that separates subsite I and II. However, ligand binding causes a 180° rotation of Met362 resulting in an open conformation that joins the two subsites allowing the ligands to occupy the entire binding pocket [8, 57]. In the free state of β-clamp, Met362 is highly flexible compared to other residues in the binding pocket [33], and molecular dynamics (MD) simulations show that the two conformations are equally populated, suggesting that Met362 does not function as a “gate” blocking binding of one of the subsites [73]; yet, its function remains unknown.

For most ligands, the conserved Gln resides in subsite II forming hydrogen bonds directly to β-clamp and via a conserved water molecule [8, 73]. The aliphatic residue in the second position binds to a shallow pocket in subsite II opposite the Gln pocket. The two C-terminal hydrophobic residues at position 4 and 5/6 dock deeply into a combined hydrophobic pocket in subsite I. Studies have shown that the LF moiety of the CBM is capable of binding β-clamp on its own [72, 73], and it has been suggested that LF bind to subsite I first and act as anchor allowing QL to subsequently bind subsite II. Furthermore, desolvation of the binding pockets play a major role in the binding process [57, 73]. Although Pol II and Pol III α C-terminal CBM peptides bind similarly to subsite II, there are substantial differences in their binding to subsite I (Fig. 5c). This is likely caused by the extra residue in the Pol III CBM [46]. A similar divergence in binding conformation is seen for all ligands shown in Fig. 5b, where the structures align in subsite II, but differ in subsite I.

Although many β-clamp ligands have similar CBMs and adopt similar conformations in the binding pocket, some diverge. For example, the δ subunit of the clamp loader complex does not contain a conserved Gln at position 1 of the CBM (AMSLF), yet its conformation in the bound state is very similar to other ligands (Fig. 5b, **purple**). The δ subunit has a Gln just prior to the first CBM residue, but this residue does not occupy the Gln pocket of β-clamp. Further, although the CBM of Pol V highly resembles those of the other ligands (QLNLF), the first Leu does not make a similar contact with subsite II of the β-clamp binding pocket as other CBMs and is more solvent exposed. Also, Phe is not located in the hydrophobic pocket at subsite I but is exposed to the solvent (Fig. 5b, **green**). Instead, Asn at position 3 forms an interaction with Arg152 of β-clamp [8]. This complements the results of Beuning et al. [74] who noticed a dramatic impact on the biological function of Pol V when Asn was mutated to Ala compared to mutations of the three hydrophobic residues. Still, the ligand deviating most is the mismatch repair protein MutL. The CBM of MutL (QPLLI) is in a structured region of its regulatory domain. Like other ligands, Leu at position 4 and Ile occupy the hydrophobic pocket of subsite I, but the conserved Gln does not bind in subsite II (Fig. 5b**, orange**). Instead, a sulphate ion occupies this site. The suggested explanation is that Pro disfavours the interaction of Gln with β-clamp because of the restricted φ/ψ-angles dictated by it [9].

In the *E*. *coli* β-clamp, Gly174 in subsite I and Val344 in subsite II are the only residues of the binding pocket that are not conserved (Fig. S3), demonstrating how the CBM binding site has been evolutionarily preserved. Indeed, β-clamps from different organisms can bind the same ligands [76–78]. Although the Pol IV CBM has similar affinities for different β-clamps, the binding affinities and thermodynamic profiles of other ligands may vary [76]. Despite structural conservation, subtle differences in the binding pockets and binding patterns between the species exist [9, 77, 79]. It has been suggested that subtle differences in the structural dynamics of the residues that form the binding pockets determine the differences in binding across species [76] and such variations may have implications for the development of drugs targeting specific β-clamps, as discussed below.

#### The effect of flanking regions and folded domains on β-clamp interaction

The interactions between β-clamp and its partners are often defined by the interaction of the CBMs. However, flanking sequence regions outside the CBM as well as contacts made from other (folded) parts of the ligand, can impact binding affinity, conformation, and function as seen for other SLiMs [80]. For the human DNA sliding clamp, PCNA, positively charged residues in the flanking regions just outside the p21 PCNA interaction motif modulate affinity by more than four orders of magnitude [23]. In this section, we therefore consider if and how additional interactions outside the CBM may influence β-clamp binding.

While interaction with β-clamp through CBMs are mainly driven by hydrophobic interactions, electrostatic interactions may be important. As visualized in Fig. 2c, the surface around the CBM binding pocket is highly positively charged compared to the rest, especially around subsite I, which also contains several arginines. Notably, the flanking regions of the CBMs are mostly negatively charged, either arising from negatively charged amino acids or from the free carboxyl of the C-terminus (Pol II, Pol IV and Pol III α). These complementary charges could serve to increase ligand affinity by increasing the association rate through electrostatic steering [81, 82]. Removing the positive charge of the N-terminus by acetylation of Gln of the consensus CBM (QLDLF) significantly increases the affinity towards β-clamp [57, 73, 75], supporting this idea. The carboxyl group of the C-terminal Leu of Pol IV is involved in an electrostatic interaction with Arg152 of the β-clamp binding pocket [7]. Similarly, the side chain of the C-terminal Asp, located just after the Pol III α C-terminal CBM, and its free carboxyl group interact with Arg246 [46]. Changing this Asp to Ala decreases the affinity for β-clamp [47]. Although the negatively charged residues immediately outside the CBM can affect binding affinity, N-terminal extensions of the C-terminal Pol III α CBM have only little effect (Table 1), and ITC data suggest that the CBM is responsible for the majority of the binding enthalpy [46]. In contrast, a Met just upstream of the Pol IV CBM (MERQLVLGL) interacts with β-clamp and is deeply buried in a non-canonical binding pocket [7], calling for a broader view.

**Fig. 6:**
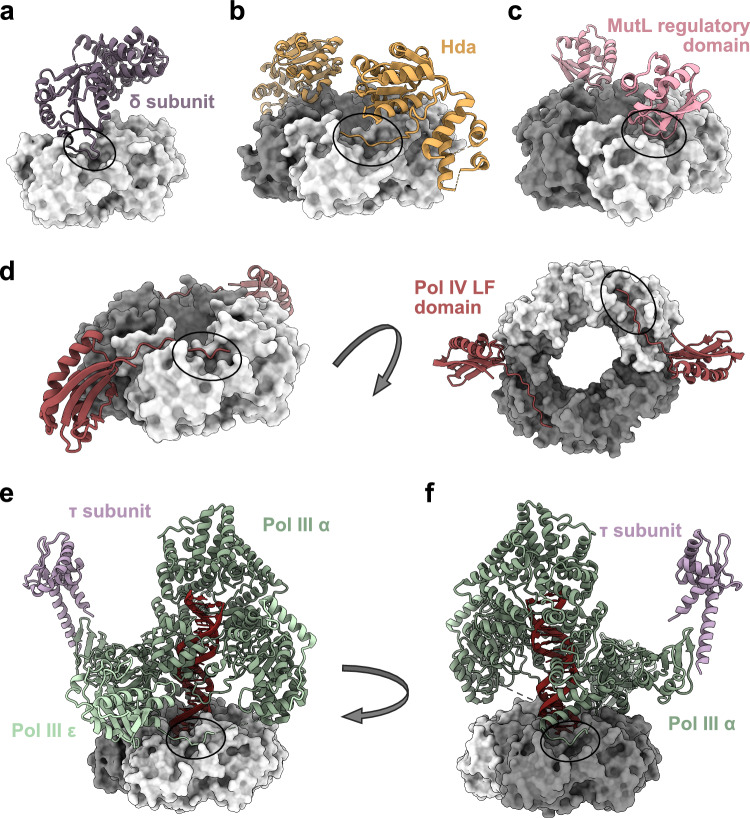
Structures of β-clamp in complexes with full-length proteins or protein domains. β-clamp is shown as dark and light grey surface, and interaction partners are shown in cartoon. **a** β-clamp Ile272Ala;Leu273Ala variant in complex with the δ subunit of the clamp loader complex (PDB ID: 1JQL [61]). **b** Structure of the β-clamp:Hda complex (PDB ID: 5X06 [67]). **c** β-clamp in complex with the regulatory domain (RD) of MutL (PDB ID: 6E8E [9]). In this structure, MutL was covalently fused to β-clamp due to low affinity of the complex. For simplicity, we have chosen not to show the linker between the two proteins and additional residues from the N-terminal affinity-tag. **d** Structure of β-clamp in complex with the LF domain of Pol IV (PDB ID: 1UNN [7]). **e** and **f**: Cryo-EM structure of β-clamp in complex with Pol III α, ε and the C-terminal part of π (PDB ID: 5FKV [35]), with Pol III ε binding to the canonical binding site of one subunit of β-clamp (**e)** and Pol III α binding to the binding site of the other subunit of β-clamp (**f**)

Structures of β-clamp in complex with full-length proteins/protein domains are available (Fig. 6a–d) including a cryo-EM structure of β-clamp in complex with both Pol III α, ε, and including the C-terminal part of π, which binds to the α subunit (Fig. 6e, f). The structures illustrate different ways by which these protein ligands interact with β-clamp. While the δ subunit of the clamp loader complex primarily interacts with the canonical binding pocket of β-clamp (Fig. 6a), Hda and the regulatory domain of MutL make additional contacts to the β-clamp surface (Fig. 6b, c). Most notably, the disordered C-terminal tail of the little finger (LF) domain of Pol IV interacts with a large surface area of domain III of β-clamp, positioning a neighbouring folded domain at the interface between the two β-clamp subunits (Fig. 6d). Figure S3 illustrates how these additional contacts to β-clamp outside the canonical binding pockets are highly unique for the different interaction partners. Interestingly, the cryo-EM structure of the β-clamp:Pol III complex shows that the α and ε subunits of Pol III each bind to separate subunits in the β-clamp dimer, while also making additional contacts to the β-clamp surface (Fig. 6e, f). These structures illustrate one reason why the entire Pol side of β-clamp, and not just the canonical binding pocket, is highly conserved (Fig. 4).

Binding of CBM peptides vs. full-length proteins has implications for affinities. The isolated CBM of Pol II and the C-terminal CBM of Pol III α each have lower affinities towards β-clamp compared to their full-length versions (1.7 µM versus ~30–120 nM and 1.4 µM versus ~0.1–1.5 µM, respectively, Table 1) [47]. While the internal CBM of Pol III α stabilizes the interaction, an oligonucleotide/oligosaccharide binding (OB) domain of Pol III α forms additional contacts with β-clamp [35] and enhances affinity > 7 fold [50]. The fact that the CBM of the δ subunit is poor at outcompeting binding of full-length δ suggests that the interaction is driven by other contacts than with the CBM alone. Indeed, the key interacting residues of the δ CBM (M, L and F) only account for 44% of the buried surface area upon complex formation [49]. Similarly, full-length Pol IV forms stronger interaction with β-clamp compared to its CBM only (~2 µM versus 465 nM, Table 1), owing to the additional contacts made by the rest of the LF domain. These additional interactions participate in the stabilization of the β-clamp:DNA:Pol IV complex [83].

Affinity measurements from pair-wise protein:protein interactions may not reflect the *in vivo* situation since many β-clamp binding partners form larger complexes. For example, the binding of β-clamp to the α subunit of Pol III has a *K*_D_ in the low micromolar range (Table 1), whereas the affinity of the entire Pol III core complex is ~50 nM [47, 48], suggesting that ε strengthens the interaction of the α subunit with β-clamp. Stabilization by the exonuclease was also investigated by Toste Rêgo et al*.* [54] who found that ε increased the affinity ~4 fold. Furthermore, when β-clamp is bound to DNA, its affinity for the Pol III core complex is considerably increased (*K*_D_ < 5 nM) [14]. There is also a slight increase in binding affinity of the full γ complex compared to binding of δ alone [62]. The structures of β-clamp with full-length protein or protein domains can provide additional functional information. For example, β-clamp in complex with the LF domain of Pol IV show Pol IV to be inaccessible to DNA and the authors therefore suggest that Pol IV is maintained in an inactive state by the additional interactions to the β-clamp dimer interface [7]. An additional non-canonical binding interface of the β-clamp:Hda complex has been shown to be important for the oligomerization of Hda and for its biological function in the regulatory inactivation of DnaA [67]. In contrast, residues of MutL that stabilize an additional interaction between β-clamp and MutL are not required for MutL to functionally interact with β-clamp during mismatch repair [9].

In conclusion, the interactions between β-clamp and its protein partners cannot simply be described by the interaction of the CBM alone. Electrostatic interactions of the flanking regions outside the CBMs and additional contacts formed by other parts of the folded protein ligands can modulate both affinity and function. The complete assembly of protein complexes, such as the Pol III core complex, may also modulate binding. Finally, how the binding of these full-length protein complexes is influenced by the interaction with DNA is still elusive but is likely to affect both affinities and binding conformations.

#### β-clamp differentially binds ssDNA and dsDNA

**Fig. 7:**
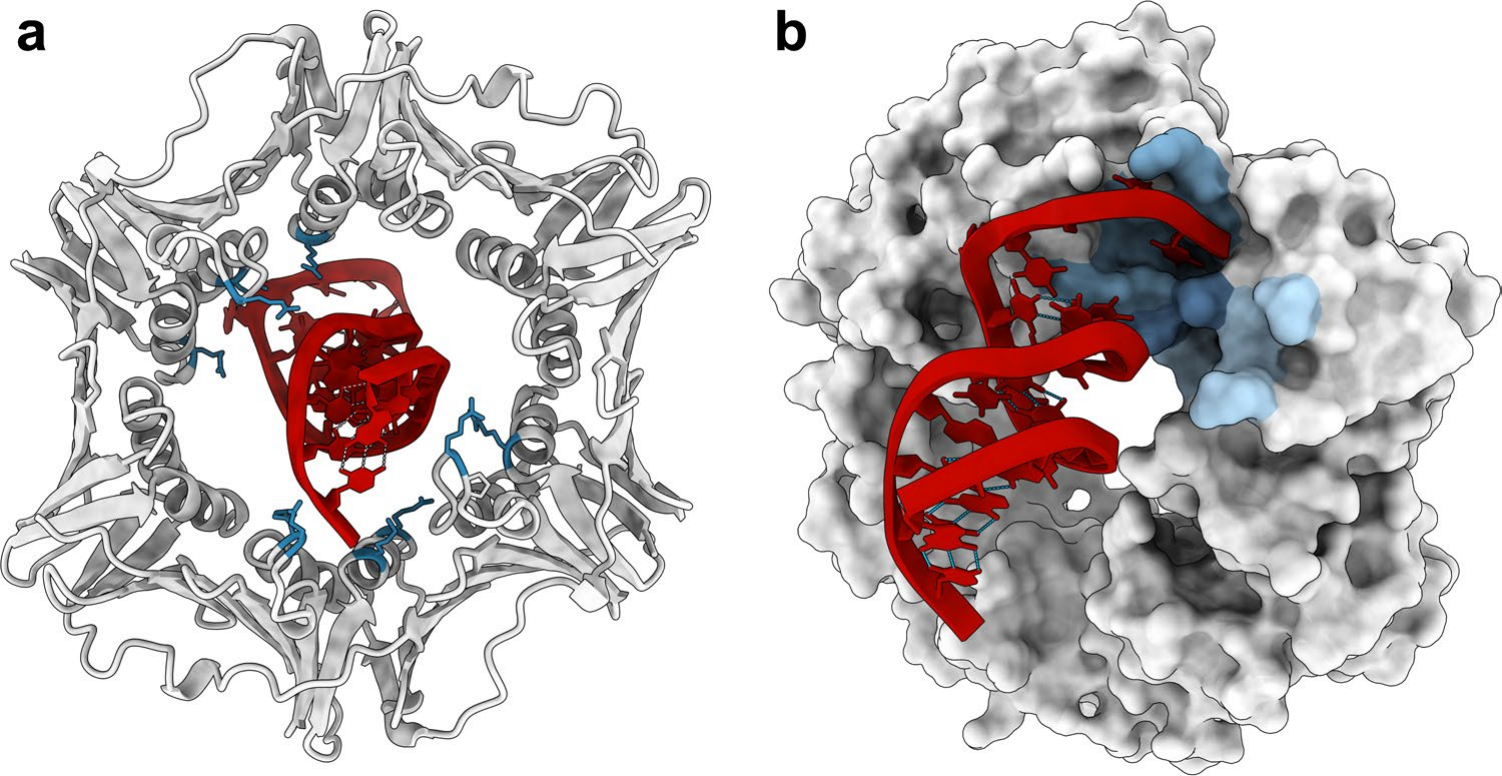
β-clamp has two DNA binding sites. **a** DNA binding in the central channel of β-clamp with the dsDNA interacting residues highlighted as blue sticks. **b** The ssDNA part of the primed DNA interacts with the canonical protein binding pocket of an adjacent β-clamp molecule in the crystal lattice. The protein binding pocket is coloured according to the two subsites using the same colouring as in Fig. 6. PDB ID: 3BEP [5]

A monomeric β-clamp variant (Ile272Ala;Leu273Ala) still binds DNA [5]. However, this monomeric β-clamp variant cannot tether Pol III to DNA for processive replication [41], implicating that the circular shape of β-clamp is crucial for biological activities. Dimeric β-clamp cannot bind circular DNA in the absence of a clamp loader, implying that DNA must bind through the central channel of β-clamp. Even though β-clamp needs a clamp loader to bind DNA *in vivo* and is not thought to make specific interactions with DNA, β-clamp can bind short fragments of linear DNA in vitro and exhibit binding saturation [5]. Georgescu et al*.* published the crystal structure of DNA-bound β-clamp [5]. The DNA is primed DNA and thus contains a dsDNA part and a ssDNA part. In this structure, dsDNA makes several contacts to residues that line the inner ring of β-clamp (Fig. 7a), including Arg24 and Gln149 located on protruding loops on the Pol side of β-clamp [5]. Since β-clamp must slide along DNA during replication, these interactions are thought to be transient. Almost all residues interacting with DNA in the inner ring are conserved (Fig. S3), supporting their functional importance.

Binding experiments reveal an affinity difference between dsDNA and primed DNA with primed DNA binding ~4 fold tighter (Table 1), indicating that β-clamp has an additional binding site for ssDNA [5]. This was further supported by the crystal structure where the ssDNA part binds in the canonical CBM binding pocket of an adjacent β-clamp molecule (Fig. 7b and Fig. S3). The authors suggest this interaction to be intramolecular in solution. Indeed, this ssDNA:β-clamp interaction can be outcompeted by a Pol III peptide, demonstrating that polymerases can compete with primed DNA for binding to β-clamp. Georgescu et al. [5] suggest that this interaction serves to keep β-clamp at the primed site where it is needed for replication, until the interaction is disrupted by polymerases, allowing β-clamp to diffuse along DNA. Single-molecule fluorescence spectroscopy experiments support this and show that β-clamp is held stationary at the primer/template (p/t) junction of primed DNA [36].

### Polymerase switching

The β-clamp is obviously involved in many different biological processes, including DNA replication, DNA repair and DNA damage control (TLS), and a fine-tuned balance between the many interactions must exist. The β-clamp dimer contains two binding sites that could be simultaneously occupied by two different proteins. Elucidating the functional outcome of β-clamp:ligand complexes is further complicated by β-clamp interacting with larger protein complexes that contain several CBMs. We will now briefly address the complexity of the β-clamp:Pol III core interaction and discuss some of the theories and models put forward on how the replicative polymerase III and the TLS polymerase IV undergo switching on DNA upon a lesion encounter.

#### The complexity and dynamics of the pol III α:β-clamp interaction

The interaction between the Pol III core complex and β-clamp is modulated by an interplay of different CBMs and interactions through different domains. Pol III α has two CBMs, an internal and a C-terminal CBM, both of which bind to the canonical binding pocket with micromolar affinity (Table 1). Furthermore, the exonuclease domain ε, also contains a CBM shown to stabilize the β-clamp:Pol III interaction [54], as described above. Some disagreement about the importance of the two Pol III α CBMs exists. Some studies find the C-terminal CBM to be important for the functional interaction of α with β-clamp [47, 48] as its removal reduces the affinity of the Pol III core (α, ε, θ) from ~50 nM to > 1 µM [48]. Similarly, its removal reduces the affinity between Pol III α and β-clamp ~10 fold [47]. In contrast, others find the internal CBM to be more important than the C-terminal CBM [51, 54, 55]. Lamers et al*.* [50] show that deletion of the C-terminal CBM of α does not affect the affinity of α to β-clamp, but reduces the processivity of DNA synthesis. The C-terminal CBM of α also binds τ, and τ can compete with β-clamp for binding the C-terminal CBM [48, 51]. Therefore, interaction with τ may be an important function of the C-terminal CBM of α *in vivo* [51]. Although the C-terminal α CBM binds β-clamp stronger than the internal CBM when tested separately [46], mutations in the internal CBM may appear more critical [51] due to the full-length protein context.

The two CBMs of Pol III α could potentially occupy both β-clamp binding sites simultaneously. However, during DNA replication, the two sites are likely occupied by the internal Pol III α CBM and the ε CBM alternating between two states; one ensuring efficient and processive DNA synthesis and the other ensuring fidelity by the exonuclease [55]. In the cryo-EM structure of β-clamp in complex with Pol III α, ε, the C-terminal region of τ and DNA (Fig. 6 e and f), the β-clamp binding pockets are indeed occupied by the internal α CBM and ε, which is wedged between the α subunit and β-clamp [35]. In particular, the OB domain of α, that stabilizes the β-clamp:Pol III α interaction [50], interacts with the surface of β-clamp. The C-terminal CBM of α is located far from β-clamp but close to the binding site with τ. However, to achieve a more stable complex, both the internal α and ε CBMs were in this study mutated into consensus-like CBMs with increased affinity [35]. It is therefore difficult to conclude whether the C-terminal CBM could interact with β-clamp, even transiently. Interestingly, in the absence of the C-terminal τ protein, the entire C-terminal domain of α including the C-terminal CBM is invisible in the electron density map due to heterogeneity [35], indicating this region of α to be highly dynamic in the absence of τ. In the DNA-free structure, the complex undergoes large conformational changes where the tail of the α subunit moves away from β-clamp, and the OB domain of α no longer makes contacts [35]. Here, the mutated internal CBMs of α and ε still bind the canonical binding pockets of β-clamp, but one might speculate that the observed dynamics could displace one or more of the subunits of the Pol III core complex. The presence of multiple CBMs in one binding partner can also increase the apparent affinity by increasing the local concentration *via* an avidity effect [84, 85]. This effect is seen in several PCNA binding proteins that contain two or more PIP boxes/degrons and/or APIM motifs [26, 86–88]. The roles of having multiple PIP motifs in Pol δ, Pol κ and Lig1 is discussed in a recent review [89], where it is proposed that ancillary PIPs function as flexible tethers. Initial binding of these ancillary PIP motifs mediates fast recruitment of these enzymes to PCNA and will be displaced by internal PIP motifs located close to the catalytic domains, allowing for a functional positioning of the enzymes on PCNA [89].

#### Polymerase switching by the toolbelt model

How do different polymerases exchange on β-clamp to ensure fast, efficient, and high-fidelity replication of the DNA, while overcoming potential DNA lesions? Because the β-clamp dimer contains two binding sites, the ‘toolbelt’ model, in which two polymerases bind the same β-clamp simultaneously, has been suggested [90]. In this model, access to the DNA template is highly regulated ensuring high fidelity replication and minimizing error-prone TLS. Studies have shown that Pol III and Pol IV can occupy β-clamp simultaneously during active DNA replication thereby possibly enabling rapid bypass of DNA lesions [91, 92]. When Pol III stalls, Pol IV efficiently binds to DNA, while Pol III remains bound to β-clamp [91] adopting an inactive conformation [92]. When the DNA lesion is bypassed, Pol III quickly regains access to DNA. Thus, the access of Pol IV to the primed site is highly regulated and only allows the low-fidelity Pol IV to access the DNA long enough to bypass the DNA lesion [91]. This regulation may be affected by the inactive state of Pol IV observed in the crystal structure of β-clamp in complex with the LF domain of Pol IV (Fig. 6d). Pol IV can then bind to β-clamp near the site of DNA replication, where it stays inactive until needed [7, 83]. Although the switching between Pol III and Pol IV in vitro has been elucidated to some extent, the model is likely simplified as β-clamp has many more interaction partners that compete for binding, especially after DNA damage induces the global SOS response. Further, when the replication fork is blocked by DNA damage it skips over the lesion and re-initiates replication downstream, leaving a ssDNA gap. The DNA damage in the gaps is next bypassed by TLS polymerases followed by a replicative polymerase filling the gap in a post-replicative manner (reviewed in [93]). Competition of TLS polymerases for β-clamp will be further modulated by the expression levels of the different interaction partners and their availability at DNA [94]. For example, the expression of TLS polymerases increases during SOS [93], likely enhancing binding to β-clamp. It is also possible that binding affinity is regulated through posttranslational modifications on the β-clamp or its binding partners, as seen in eukaryotic systems. For example, phosphorylation of the PCNA binding partner p21 regulates both degradation of p21 and binding to PCNA [95–97]. Some post-translational modifications such as lysine acetylation or ubiquitylation-like modifications (pupylation) have been detected in prokaryotes [98, 99] and may contribute to regulation. However, post-translational modifications on β-clamp have not been greatly explored.

In conclusion, many different factors likely participate in coordinating and regulating the functional hierarchy between β-clamp and its binding partners. However, more studies are needed to fully establish how these binding events are coordinated and how they are influenced by posttranslational modifications and binding to DNA.

### β-clamp dynamics and the clamp loading mechanism

Only one of the dimer interfaces needs to open for β-clamp to be loaded onto DNA [41, 63, 64, 100]. Yet, whether the clamp loader actively wrenches the β-clamp dimer open or if it traps an already transiently open conformation of β-clamp has been debated [42, 61, 101, 102]. While PCNA is a stable trimer in solution [38] and is actively opened before being loaded onto DNA [103], the T4 sliding clamp quaternary structure is less stable [38] and already adopts an open conformation in solution [104–106]. Here, we address the intrinsic dynamics of β-clamp, examine the interaction between β-clamp and the clamp loader complex and describe the clamp loading mechanism.

#### β-clamp is dynamic

Several studies have shown β-clamp to be dynamic, which may be relevant for its opening by the clamp loader. First, analysing the small differences among the β-clamp subunits in a crystal structure reveals some asymmetry. Although the differences are generally subtle and less than 1 Å [33], they are greater for domains II and III than for domain I reflecting increased dynamics and flexibility within these domains. Fang et al. [21, 42] used hydrogen-deuterium exchange mass spectrometry (HDX-MS) to study β-clamp dynamics in solution. Here they noted that despite nearly identical 3D structures, the dynamics of the three domains differ substantially. Especially at earlier time-points, domain I experience more deuterium exchange than the other domains, indicating it to be more dynamic [21, 42]. The flexibility of domain I suggests that the β-clamp dimer may open stochastically in solution, where the function of the clamp loader subunit δ could be to bind and stabilize a transiently open state of β-clamp or to further open it [42]. The hypothesis that β-clamp exists in an open-closed equilibrium has been tested using several different techniques including small-angle X-ray scattering (SAXS) data, fluorescence correlation spectroscopy (FCS) and MD simulations, which show that β-clamp mainly exists as a stable dimer [44, 61, 104, 107]. However, the opening and closing may happen at certain time-scales or at a low population, undetectable by these techniques. Finally, the inherent dynamics of β-clamp could be important for mediating interactions between different binding partners allowing binding-induced allosteric conformational changes in the adjacent subunit, important for regulation.

#### β-clamp interacts with all five subunits of the γ complex

**Fig. 8:**
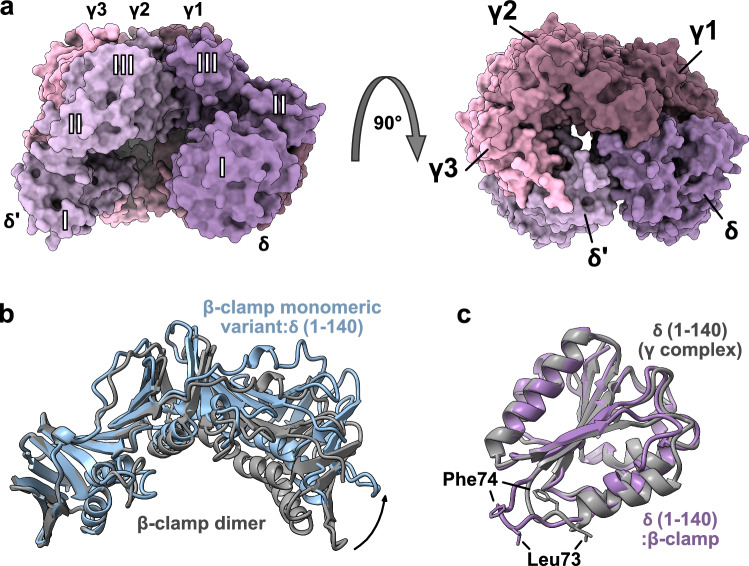
The β-clamp:δ interaction causes conformational changes in both β-clamp and δ.** a** Structure of the clamp loader γ complex (PDB ID: 1JR3 [108]) viewed from the side (left) and from above (right). **b** β-clamp monomeric Ile272Ala;Leu273Ala variant in complex with δ (1–140) (PDB ID: 1JQL [61]; δ not shown) illustrated in blue aligned with one subunit of the β-clamp dimer (PDB ID: 1MMI [33]) shown in grey. **c** Structure of δ in complex with δ′ and three γ subunits (γ1, γ2 and γ3) (PDB ID: 1JR3 [108]; only residues 1–140 are shown) shown in grey, and δ (1–140) in complex with β-clamp (PDB ID:1JQL [61]; β-clamp not shown) in purple. The complex between the β-clamp monomeric Ile272Ala;Leu273Ala variant and δ (1-140) is illustrated in Fig. 6a

An important step in clamp loading is the interaction between β-clamp and the clamp loader complex. Of the five different proteins, τ/γ, δ, δ′, χ and ψ, only the pentameric γ complex (τ_n_γ_(3−n)_δδ′) is needed for loading [100]. Each subunit in the γ complex has three domains (domains I, II and III). The C-terminal domains of the clamp loader subunits (domain III) pack together and form a ring-shaped collar, where the N-terminal domains (domains I) form an open structure (Fig. 8a) [108].

In isolation, β-clamp only forms a stable complex with δ [40, 62], and δ therefore forms the major contact between β-clamp and the γ complex. Multiple contacts with the other subunits are also formed [63, 64], although weaker [40]. The affinity between β-clamp and the γ complex is ~2–3 times stronger than between β-clamp and δ alone (7–10 nM *vs*. 3.2 nM, Table 1) [62], reflecting some contribution from the remaining γ complex. Only one δ subunit needs to bind the β-clamp dimer to form a stable interaction [41]. Still, β-clamp can bind two δ subunits simultaneously, albeit with negative cooperativity (Table 1), likely because of steric hindrance [53]. Although the δ subunit binds β-clamp on its own, it cannot load β-clamp onto DNA without the other γ complex subunits [100].

The structure of the δ subunit in complex with the monomeric β-clamp Ile272Ala;Leu273Ala variant has been solved [61]. The δ subunit consists of three domains, where domain I interacts with β-clamp (Fig. 6a). The interaction is mainly mediated by Met71, Leu73 and Phe74 of δ, where Leu73 and Phe74, which are critical for clamp loading activity [109], form a hydrophobic plug that docks into subsite I of β-clamp [61]. Additional contacts are formed between δ and a 5-residue loop in β-clamp [61]. Although none of these contacts involve the β-clamp dimer interface directly, interaction at the loop site distorts the conformation, causing structural rearrangement in the dimer interface of β-clamp leading to ring opening [61, 109]. Despite its importance in clamp loading, deletion of the loop only reduces the affinity of the β-clamp:γ complex by ~5 fold or less [109].

The β-clamp monomeric variant in complex with δ adopts a relaxed structure with less curvature (Fig. 8b). In the dimeric β-clamp, the helix of domain III in the dimer interface has a distorted geometry relative to a normal α-helix. In the monomeric structure this helix is undistorted, precluding formation of a well-packed interface [61]. HDX-MS experiments of the monomeric β-clamp Ile272Ala;Leu273Ala variant show a less compact structure relative to the β-clamp dimer. Hence, the more relaxed conformation of β-clamp in complex with δ may be caused by mutations or monomerization rather than a direct effect of δ binding [42]. MD simulations support this view and show the β-clamp monomer to relax into a more open structure resembling that of the monomeric variant in complex with δ [61], hinting that the β-clamp monomer populates a more open conformation even in the absence of δ. A fluorescence-based clamp opening assay shows that β-clamp and δ do not form a stable open complex [101], implying that a putative opening event by δ is only transient. Although very different binding affinities for δ to β-clamp are reported (Table 1), a direct comparison between the dimeric β-clamp and the monomeric Ile272Ala;Leu273Ala variant shows that δ binds the monomeric β-clamp variant ~50 times stronger than it binds the β-clamp dimer. This difference may reflect that some of the binding energy is manifested into opening the dimer interface [41]. Upon interacting with β-clamp, the δ subunit itself undergoes conformational changes. The key interacting residues Leu73 and Phe74 of δ are inaccessible in the absence of β-clamp. However, upon complex formation, a helix in δ partially unwinds accompanied by a rotation and translocation, which expose Leu73 and Phe74 allowing binding to subsite I of β-clamp (Fig. 8c) [61, 108].

Recently, two cryo-EM structures of β-clamp bound to the δ complex in the presence and absence of DNA were published [63, 64]. These reveal that all five subunits of the γ complex are in contact with β-clamp during the loading process. Here, δ binds the canonical binding site of one subunit, mediated by Leu73 and Phe74 of the known CBM, in accordance with the crystal structure of the δ:β-clamp complex [61]. γ3 binds to the canonical binding site of the other subunit mainly through the sequence 109-DNVQYAPAR, where Tyr113 serves as anchor and residues upstream contribute to binding. The same Tyr of γ1 and γ2 also serve as anchor for binding into different pockets on β-clamp [63]. The γ1 subunit binds β-clamp between domain I and II, while γ2 mainly binds to domain I near the closed dimer interface. δ‘ binds between domain I and II of the other β-clamp subunit [63, 64]. Although all subunits of the γ complex contact β-clamp, the CBM interaction of δ fits better into the binding site on β-clamp, and the binding interface is more hydrophobic, whereas the other interactions are more hydrophilic and weaker [63]. The δ:β-clamp interaction therefore contributes with more binding energy than the binding interfaces of the other clamp loader subunits [63], consistent with previous findings.

#### ATP binding and hydrolysis drives clamp opening and loading onto DNA

ATP binding is critical for loading β-clamp onto DNA [110, 111]. The γ complex contains three ATP binding sites located at the γ1–γ2, γ2–γ3 and γ3–δ′ interfaces [63]. The interaction with β-clamp is highly ATP dependent; the presence of ATP increases the affinity for the γ complex towards β-clamp by three orders of magnitude (Table 1) [62]. In contrast, δ binding to β-clamp is not ATP dependent and its affinity is similar to that of the γ complex in the presence of ATP [62]. Although it was previously proposed that the β-clamp interacting residues of δ would be inaccessible in the γ complex in the absence of ATP, the CBM of δ is solvent exposed in crystal structures of the γ complex in both the absence and presence of bound nucleotides or nucleotide analogues [108, 112]. The conformational changes in the γ complex triggered by ATP binding is therefore likely a more complex mechanism, where ATP binding causes cooperative rearrangements in the clamp loader resulting in an altered conformation compatible with β-clamp binding [111, 112]. This ATP-dependent conformational rearrangement necessary for β-clamp binding is at least partially driven by the requirement of δ′ to promote these changes [109].

ATP binding to the γ complex is important for clamp opening [100, 101]. In the absence of DNA, β-clamp suppresses ATP hydrolysis by the γ complex, whereas the presence of both β-clamp and primed DNA enhances both ATP hydrolysis and β-clamp closing and release ~200 fold [100, 113]. This suggests a mechanism that prevents ATP hydrolysis and closing of the β-clamp until bound to DNA. Both ATP binding to the γ complex and the ATP-induced conformational changes important for β-clamp binding occur relatively fast. However, another set of ATP-induced conformational changes promote binding to DNA and this process occurs slower and is likely rate limiting [114]. These two different types of ATP-induced conformational changes occurring at different timescales could serve as a mechanism where the clamp loader favours β-clamp binding before DNA binding, ensuring an efficient loading cycle.

#### The clamp loader opens β-clamp in a crab-claw-like motion

**Fig. 9:**
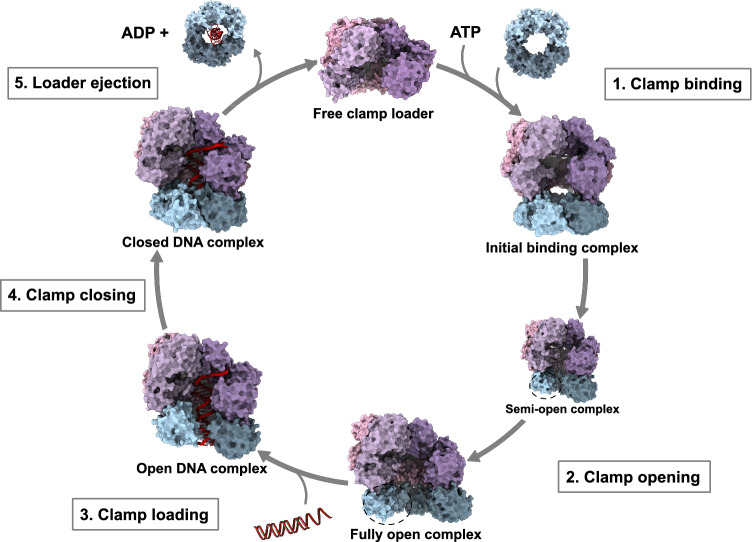
Clamp loading mechanism. The cryo-EM structures of the β-clamp:clamp loader complex in this figure are the work of Landeck et al. [64]. 1. Free clamp loader (PDB ID: 1JR3 [108]) binds ATP and then β-clamp (PDB ID: 1MMI [33]) to form the initial binding complex. 2. The clamp loader complex then partially opens β-clamp before it forms a fully open complex. Dashed circles indicate parts of β-clamp with missing density. 3. Primed DNA binds the central channel of the β-clamp:clamp loader complex. 4. β-clamp closes around DNA, and the contacts with the clamp loader becomes less pronounced. 5. The clamp loader dissociates from β-clamp that is left behind on DNA (PDB ID: 3BEP [5]) and the clamp loader can then re-bind ATP and be used for another clamp loading cycle. ATP hydrolysis may occur prior to clamp closing and ejection. This figure is inspired by the work and figures of Xu et al. [63] and Landeck et al. [64]. The cryo-EM structures by Xu et al. [63] were not available at the time this review was submitted

How the clamp loader complex opens β-clamp prior to loading has been greatly debated. While some papers suggest that the clamp loader actively opens β-clamp at the dimer interface [101, 102], other found indications that the clamp loader traps an already open β-clamp conformation [42, 61]. The recent cryo-EM structures of β-clamp bound to the γ complex in the presence and absence of DNA have provided new insights. Both studies found that the γ complex actively opens β-clamp using a crab-claw-like mechanism led by structural rearrangements within the clamp loader complex opening the β-clamp dimer interface wide enough for DNA to enter [63, 64]. The structures can be assigned to four different steps of the clamp loading mechanism (Fig. 9).

- **1) The initial binding complex**: The clamp loader first binds β-clamp *via* the canonical CBM of δ subsequently assisted by the other γ complex subunits [63]. The clamp loader binds a closed β-clamp, mainly mediated by δ and γ3 and to a lesser extent by γ1 [63, 64]. Xu et al. [63] saw that δ′ only makes minimal contact, while Landeck et al. [64] found substantial contacts between δ′ and β-clamp in the closed state. The closed conformation is a mixture of considerably different conformations, reflecting the dynamic nature of clamp binding [63]. The clamp loader adopts a conformation that prevents ATP hydrolysis [63, 64].
- **2) Opening of β-clamp**: During clamp opening, conformational changes, in which the γ1:γ2 interface becomes tightly packed, drive δ and γ1 to rotate outwards. Since γ3 binds the opposite binding site on β-clamp, this rotation pulls the dimer interface apart. At least two open conformations of clamp loader bound β-clamp are seen [63, 64]. In the semi-open complex, β-clamp adopts a spiral conformation [64], whereas in the fully open complex β-clamp is planar with a ~20 Å gap, wide enough for dsDNA to enter the central ring [63]. The largest conformational changes occur in domain I and II in one β-clamp subunit, whereas the changes in the other subunit are more subtle, except for domain I [63, 64]. In one study there was no density from domain I at the opening site, suggesting a highly dynamic open structure [64], which is further supported by HDX-MS data, where cooperative unfolding of domain I is observed [42]. In the other study, domain I is resolved, but appears flexible and adopts a more extended structure resulting from the released tension of the closed β-clamp dimer interface [63]. In the open complex, all five subunits of the γ complex contact β-clamp more extensively relative to the closed state [63, 64]. Kinetic data on clamp binding and opening also suggest two open conformations of β-clamp bound to the clamp loader, with the first opening occurring relatively fast followed by a slower conformational rearrangement of the open complex [102], as visualized in the cryo-EM structures.
- **3) DNA binding**: Cryo-EM structures of the β-clamp:γ complex bound to primed DNA reveals that the initial interaction with DNA is mainly mediated by the clamp loader, which binds to template DNA, while β-clamp only makes limited contact to it [63]. In the open β-clamp:clamp loader:DNA complex, the γ complex and β-clamp adopt a spiral conformation that matches the helicity of DNA. Both studies identified two similar open states of this ternary complex, likely representing intermediate structures before the clamp closing event [63, 64].
- **4) β-clamp closing around DNA**: In the closed β-clamp:clamp loader:DNA complex, the β-clamp structure is essentially similar to the unbound state [64], except that domain I may be slightly extended as seen in the β-clamp:δ complex [63]. Closing of β-clamp around DNA is only associated with very subtle conformational changes in the clamp loader complex, but during β-clamp closing, the γ complex, particularly γ2, γ3 and δ′, lose interactions to β-clamp [63, 64]. In the closed ternary complex, β-clamp directly binds to DNA mediated by Gln15, Arg73, Arg80 of one subunit and Gly23, Arg24, Arg80 and Gln149 of the other subunit [64], and most of these residues also interact with dsDNA in the absence of the clamp loader complex (Fig. S3) [5].
- Mutations of some of the ssDNA and dsDNA interacting residues in β-clamp reduce the replication activity due to defects in clamp loading. These mutations do not greatly affect the affinity for the clamp loader, suggesting an importance in a clamp loading step after the initial formation of the β-clamp:clamp loader complex. Mutations of some of the dsDNA interacting residues also impair clamp loading due to reduced β-clamp:DNA interactions [6]. Georgescu et al. [5] propose that once the clamp loader positions β-clamp around DNA, the attractive interactions with DNA induce β-clamp to close around DNA. Closing the β-clamp ring to form planar structure may thus weaken the interaction with the spiral-shaped clamp loader complex. Following this destabilization, ssDNA at the primed site may displace the δ subunit at the CBM pocket releasing the clamp loader complex.
- **5) Loader ejection:** ATP hydrolysis is important for clamp release [100, 110] and this hydrolysis happens in a sequential order [115]. However, the exact role of ATP hydrolysis in clamp release is still not clearly understood. While fluorescent assays suggest that ATP is hydrolysed before β-clamp closes around DNA [113], structures of the closed β-clamp:clamp loader:DNA complex indicate that ATP hydrolysis may not be necessary for clamp closing, but rather drives clamp loader dissociation [64].

#### The δ subunit functions as a clamp unloader

Besides its function in clamp loading, the γ complex also serves as a clamp unloader in an ATP-dependent mechanism [14, 53]. While the β-clamp itself remains relatively stable when bound to DNA, the γ complex catalyses fast unloading of β-clamp with a half-life of ~88 s [13]. Although the entire γ complex is necessary for clamp loading, the δ subunit is just as efficient at unloading β-clamp from DNA as the γ complex, and unloading by δ does not require ATP [13, 53, 100]. In *E. coli*, δ is present at 5–7 fold excess over the γ complex, hinting that δ alone may be responsible for β-clamp unloading *in vivo* [13]. Binding of δ may therefore provide sufficient energy to destabilize the dimer interface of β-clamp, whereas clamp loading is a more complicated process [100]. However, even though one β-clamp binding site is enough to efficiently load β-clamp onto DNA, both binding sites are required for efficient unloading of β-clamp by both the γ complex and δ alone [53], implying that the unloading mechanism may also be more complex than anticipated.

#### Molecular switching between clamp loader and polymerases

Pol III and the clamp loader compete for the same binding sites on β-clamp. How this is coordinated to ensure that the clamp loader binds β-clamp when it needs to be loaded onto DNA and leaves β-clamp to interact with Pol III after loading, remain enigmatic. Even though β-clamp has two binding sites, it does not simultaneously bind the γ complex and Pol III core [14], but prefers the γ complex over the Pol III core, consistent with the differences in affinity (~9 nM on average *vs*. ~50 nM, Table 1). However, when β-clamp is bound to DNA, the affinity between β-clamp and Pol III core increases (*K*_D_ < 5 nM), likely due to additional interactions formed between Pol III core and DNA [14]. In the absence of β-clamp, the ATP-bound γ complex binds primed DNA with nanomolar affinity, but after DNA binding and subsequent ATP hydrolysis, the γ complex is converted to an inactive state with decreased affinity (micromolar) towards DNA [116]. DNA may therefore directly mediate a molecular switch between the clamp loader and the replicative polymerase.

### β-clamp drug targeting

The emergence of antibiotic-resistant bacteria necessitates the development of antibiotics with novel targets. β-clamp is attractive in this regard as it plays a central role in essential bacterial processes. Targeting β-clamp can have dual benefits; interfering with its role in DNA replication can inhibit the ability of the bacteria to grow and survive, while interfering with its role in TLS can inhibit mutagenesis and thereby the development of antibiotic resistance. As β-clamp is an essential scaffolding protein, it makes the development of resistance less likely, since mutations affecting β-clamp-protein interactions would lead to a loss of fitness for the bacteria. A list of β-clamp targeting drugs is presented in Table 2 and will be discussed below.

**Table 2:** β-clamp targeting drugs

Drug	Class of drug^a^	Mode of action	MIC	Challenges	Stage
Betatides [31, 117, 118]	Peptide (H)	Inhibit β-clamp:protein interactions, such as polymerases	< 16 µg/mL / < 4 µM (range of bacteria species)	Effects also stress reactions in human cells	Preclinical
CBM-derived peptides [27, 73, 75, 119]	Peptide (H)	Inhibit β-clamp:protein interactions, such as polymerases	Not detected	Bacterial uptake	Preclinical
Griselimycin and derivatives [120, 121]	Peptide (H)	Inhibit β-clamp:protein interactions, such as polymerases, mainly in *Mycobacterium.*	< 1 µg/mL (*Mycobacterium* species)	Poor pharmacokinetics of griselimycin, improved for derivatives.	Preclinical
RU7 [46]	Small molecule (H)	Inhibit some Gram negative β-clamp:protein interactions, mainly Pol III	Not detected	Only in vitro activity demonstrated	Preclinical
Compound 8 [122]	Small molecule (H)	Inhibit β-clamp:protein interactions, such as polymerases	39–78 µg/mL / 156–313 µM (range of bacteria species)	Weak *in vivo* activity	Preclinical
Diflunsial [123]	Anti-inflammatory drug, repurposing (H)	Inhibit β-clamp:protein interactions, such as polymerases, mainly in *H.pylori*	84 µM (*H.pylori)*	Weak *in vivo* activity	Preclinical
NSAIDs [124]	Anti-inflammatory drug, repurposing (H)	Inhibit β-clamp:protein interactions, such as polymerases	44-1400 µg/mL / 156-5000 µM (range of bacteria species)	Weak *in vivo* activity	Preclinical
Twort/GP168 protein [78]	Bacteriophage (C)	Inhibit clamp loading on DNA causing replication arrest in selective strains (*Bacillus, Clostridium)*	Not reported		Preclinical

^a^(H) indicates that the molecule binding site is the hydrophobic pocket and (C) indicates that it binds to the DNA channel

#### Targeting β-clamp with APIM and CBM derived peptides

Selectively targeting β-clamp without affecting the structurally and functionally similar mammalian homologue, PCNA, could be challenging because there is cross-reactivity between eukaryote and prokaryote CBMs. This was discovered by chance as a result of a bacterial infection in cancer cell cultures during the development of a cell penetrating peptide containing the PCNA interacting APIM motif [31]. APIM and the PIP-box both bind to the hydrophobic pocket of PCNA [125, 126], which is functionally conserved with the CBM binding pocket on β-clamp. APIM-peptides have good antibacterial activity (minimal inhibitory concentrations (MIC) < 16 µg/ml), inhibit DNA replication and TLS, and bind directly, albeit with low affinity in vitro, to the binding pocket of β-clamp [31]. There are no co-crystallization data on the APIM:β-clamp interaction, but the second and third amino acids of the APIM (WL) are postulated to bind to subsite I, interacting mainly with Val247 and Pro242, while subsite II is proposed to bind to the N-terminus of the APIM-peptide (Ac-MD) mainly *via* Pro363 [31]. The fact that APIM interacts with β-clamp supports that its binding pocket can bind CBMs with very different compositions.

The unique feature of APIM-containing peptides is that they block replication in bacterial cells, whereas they do not affect DNA replication in eukaryotic cells, only interfering with the PCNA-protein interactions that are important during cellular stress including DNA damage [127–131]. This is because the affinity of APIM to PCNA is increased upon posttranslational modifications on PCNA [26, 88], and this limits the APIM-peptides cytotoxicity to healthy mammalian cells, as confirmed in a phase I clinical trial [28]. This property increases their potential for further development to new antibacterial drugs. Optimized APIM-containing peptides, called betatides (β-clamp targeting peptides), have low MIC against all bacterial strains, including multidrug-resistant strains listed by the World Health Organization (WHO) as particularly virulent and increasingly multidrug resistant (ESKAPE pathogens; *Enterococcus faecium, S. aureus, Klebsiella pneumoniae, Acinetobacter baumannii, Pseudomonas aeruginosa, Enterobacter* species). Importantly, betatides have no cross-resistance and are difficult to develop resistance against [117]. Betatides have also demonstrated antibacterial activity in *in vivo* preclinical infection models with no toxicity to the model mice or rats [31, 118]. These antibacterial peptides are still in preclinical phase development.

Peptides containing the consensus CBM (QLDLF) have also been investigated for antibacterial activity (reviewed in [27]). The affinity of β-clamp for these peptides can be increased 110 fold by acetylation of Gln and substitution of Phe with 3,4-dichlorophenylalanine [73, 75]. However, the ability to inhibit bacterial growth was not demonstrated for these peptides, probably due to limited bacterial uptake. Conversely, overexpression of the CBM-peptide MGPRQLDLF cloned into *E. coli* inhibits colony forming capacity and bacterial growth, supporting their potential once uptake issues are resolved. To overcome this, André et al., fused the CBM-peptide called P_7_ (Ac-QXDLF-OH) to proline-rich antimicrobial peptides (PrAMPs), which allowed uptake into *E*. *coli*. PrAMPs themselves have antibacterial activity because they target the bacterial ribosome. Although the fused peptide did not reduce MIC compared to the PrAMP alone, an *in vivo* study in *E. coli* infected *Drosophilia melanogaster* flies show increased survival of flies treated with the fused peptide compared to either peptide alone. Importantly, no toxicity or reduced survival in uninfected flies were seen, supporting a therapeutic window for these CBM-peptides [119].

#### A natural β-clamp-targeting cyclic peptide

A natural β-clamp targeting peptide with *in vivo* antibacterial activity against *Mycobacteria* is a cyclic peptide called griselimycin produced by *Streptomyces*. Griselimycin and its chemical variants have been optimized for use in the treatment of tuberculosis and are shown in mouse models to be effective against tuberculosis in combination with other drugs. The most promising variants of griselimycin bind with high affinity to the conserved hydrophobic pocket of β-clamp from *Mycobacterium smegmatis* and *M*. *tuberculosis*, but have low affinity for the *E*. *coli* β-clamp and no affinity for PCNA [120]. Their development was a collaboration between researchers at Sanofi and the TB Alliance, and one of their lead variants, cyclohexyl-gricelimycin, was later shown to also have activity against *M*. *abscessus* in mice [121]. These peptides are still in preclinical development. However, the gricelimycin results show that some β-clamp-targeting peptides may have a narrower specificity, despite β-clamp being so conserved. These peptides are therefore likely to have low affinity for PCNA, advantageous for use in mammals.

#### Targeting β-clamp using small molecule binders

Small molecules that bind to and inhibit β-clamp functions have been identified, and they generally bind to a smaller area in the hydrophobic pocket than CBM peptides. The small molecule RU7 inhibits Pol III activity in vitro without affecting the eukaryotic replicative polymerase Pol δ [46]. However, RU7 has yet to demonstrate antibacterial activity *in vivo*. The small molecule derived from tetrahydrocarbazole called Compound 8 also binds β-clamp and blocks its polymerase interactions, thereby inhibiting DNA replication in vitro. Compound 8 has weak antibacterial activity in both Gram-positive and Gram-negative bacteria, but only at high micromolar concentrations [122]. The low activity of these small molecules may be due to low bacterial uptake, weaker affinity, or, as discussed below, that the *in vivo* situation is different from in vitro.

Several efforts have been made to screen already approved drugs for β-clamp binding. For example, the anti-inflammatory drug diflunisal was found by co-crystallization to interact with almost all residues in subsite I of the binding pocket of β-clamp from *H*. *pylori*, and to kill *H*. *pylori* in the high micromolar range (MIC of 84 µM) [123]. Based on computational docking scores on the *H*. *pylori* β-clamp, the same group later examined three molecules (5-chloroisatin, carprofen, and 3,4-difluorobenzamide) and found by co-crystallization that these also bound in subsite I [77]. However, only 5-chloroisatin and 3,4-difluorobenzamide had antibacterial activity, with the lowest MIC of 18 µM and less inhibitory effect in *E. coli* [123]. In addition, several non-steroidal anti-inflammatory drugs (NSAIDs) have weak antibacterial activity, bind to subsite I of the binding pocket in the *E. coli* β-clamp and interfere with DNA replication in vitro [124]. However, the NSAIDs only inhibit bacterial growth at very high concentrations (MIC ≥ 2500 µM).

#### The future of β-clamp targeting antibiotics

The focus on targeting β-clamp has largely been directed against the main protein-interacting site, i.e., the canonical binding pocket discussed in this paper. Recently, a suggested alternative strategy might be to exploit the mechanisms used by bacteriophages to inhibit β-clamp. For example, when infecting *S. aureus,* the bacteriophage Twort releases the Gp168 protein, which binds to β-clamp and prevents it from being loaded onto DNA. The target site of Gp168 is not the canonical binding pocket but the DNA channel [78]. The target site is conserved in some bacterial species, such as *Bacillus* and *Clostridium* strains, but not in *E. coli*, suggesting that Gp168 activity is specific for selected bacteria. The channel is a potential new target site on β-clamp that should be further investigated. Recently, peptide-based covalent inhibitors reacting with the conserved His175 residue of the CBM binding pocket has also been explored [132].

Peptides containing CBM variants as well as different small molecules all target the β-clamp binding pocket. However, despite their ability to interact with the β-clamp, their antibacterial activity is highly variable and not directly related to their in vitro affinities. This functional variability may be due to low bacterial uptake and/or the complexity of the *in vivo* situation where β-clamp engages in large protein complexes. This may limit their ability to block β-clamp interaction partners and thus their antibacterial activity. Is β-clamp promiscuous or are the interactions tightly regulated? For PCNA, the latter appears to be the case. Motifs like APIM or PIP box CBMs are found in more than 600 different proteins [26, 133], and their affinity is thought to be regulated at least by post-translational modification of PCNA, but also of the ligands themselves as seen for example for p21 [96, 97]. Since β-clamp have multiple binding partners and is found in larger complexes, an additional layer of regulation may involve binding/stabilization from additional binding partners in these complexes. There are much fewer binding partners identified to bind β-clamp compared to the menagerie identified to bind PCNA. However, since DNA sliding clamps are functionally conserved, only a subset of the actual β-clamp binding partners may have been identified. Therefore, the consensus CBM for β-clamp may represent a limited view on β-clamp binding. The APIM CBM has low affinity for β-clamp in vitro, but APIM-peptides inhibit replication and TLS and have a low MIC on several bacterial species *in vivo* [31]. This suggests that other factors, such as post-translational modifications, additional interacting proteins/protein complexes and thus, the broader conformational landscape surrounding β-clamp guide *in vivo* interactions. A better understanding of the roles of β-clamp, its posttranslational modifications, its interaction partners, and their regulation, is crucial for designing novel β-clamp targeting compounds as antibiotics for the future.

### Outlook

The β-clamp constitutes a central hub in DNA replication, DNA repair and DNA damage avoidance and serves as a promising drug-target for antimicrobial drugs. Although much effort has been put into understanding the structure, dynamics and interactions of the *E. coli* β-clamp, many questions remain unanswered. The current definition of the β-clamp CBM may be too narrow, and like its conserved ortholog PCNA, many more interaction partners likely exist that are yet to be discovered. Looking for other binding motifs, including the APIM motif or variations of the canonical CBM may help. Furthermore, looking beyond the CBM pocket and exploiting other sites on β-clamp for drug targeting, such as the DNA channel, are relevant to consider.

The structural and functional effect of having several CBMs present in one binding partner or binding complex interacting with β-clamp is also not understood, hindering a full understanding of the biological processes mediated by β-clamp. Again, multiple CBMs are seen in several PCNA binding partners, offering additional mechanisms as avidity, allovalency and balanced affinities [22]. Combining these many options with a palette of possible posttranslational modifications, a highly understudied area for β-clamp, will have a huge potential to broaden the view on β-clamp function and guide its targeting. It is still unclear how β-clamp balances and orchestrates its interactions for example with respect to replicative and TLS polymerases during different stages of cell cycle progression and cellular stress. A functional hierarchy cannot just be a matter of binding affinities, but is likely regulated by ligand availability, expression levels and posttranslational modifications of both β-clamp and its partners, as seen for PCNA. DNA may play critical roles in regulating these interactions, both by modulating affinities and causing conformational changes. Yet, how DNA binding and DNA lesions influence selection of interactions with β-clamp remain undisclosed.

The unanswered questions regarding regulation of β-clamp:protein interactions could have implications for development of new antimicrobial drugs. Targeting β-clamp with APIM containing peptides demonstrates that we cannot yet correlate in vitro binding and affinity with *in vivo* antibacterial activities. It is possible that the broad conformational landscape allowing a diversity of β-clamp states is influenced by ligand binding and modification, further highlighting the importance of focused research within this area.

### Supplementary Information

Below is the link to the electronic supplementary material.

Supplementary file1 (PDF 575 KB)

## Data Availability

Not applicable
