# Supplementary material for: The bacterial DNA sliding clamp, β-clamp: structure, interactions, dynamics and drug discovery
Source: Cell Mol Life Sci. 2024 May 30;81(1):245. doi: 10.1007/s00018-024-05252-w (PMC11139829; doi:10.1007/s00018-024-05252-w)
Supplement: Supplementary file 1 — Supplementary file1 (PDF 575 KB) [file 18_2024_5252_MOESM1_ESM.pdf]

SUPPLEMENTARY INFORMATION

The bacterial DNA sliding clamp, β-clamp: Structure, interactions, dynamics and drug discovery

Signe Simonsen[1,2], Caroline K. Søgaard[3], Johan G. Olsen[1,2,4], Marit Otterlei[3*], Birthe B. Kragelund[1,2,4*]

[1]Linderstrøm-Lang Centre for Protein Science, [2]Structural Biology and NMR Laboratory and [4]REPIN, Department of Biology, University of Copenhagen, Ole Maaløes Vej 5, 2200-Copenhagen N, Denmark
[3]Department of Clinical and Molecular Medicine, Norwegian University of Science and Technology (NTNU), Trondheim, Norway

Corresponding author: bbk@bio.ku.dk
Correspondence also to: marit.otterlei@ntnu.no

Supplementary figures and tables

Table S1: Structural alignment of β-clamps from different organisms[a]

PDB ID	Organism	Sequence identity	RMSD (Å)[b]	Sequence length
1MMI [33]	*Escherichia coli* (Gram-negative)	-	-	366
6AMQ [134]	*Enterobacter cloacae* (Gram-negative)	96%	0.97	366
6AMS [134]	*Pseudomonas aeruginosa* (Gram-negative)	56%	1.47	367
6AP4 [134]	*Acinetobacter baumannii* (Gram-negative)	46%	1.79	388
3P16 [135]	*Mycobacterium tuberculosis* (Gram-positive)	27%	1.96	408
4TR6 [76]	*Bacillus subtilis* (Gram-positive)	27%	1.85	380
2AVT [136]	*Streptococcus pyogenes* (Gram-positive)	23%	1.94	378
4S3I [79]	*Helicobacter pylori* (Gram-negative)	20%	1.92	384

[a]Alignments were done using the TM-Align algorithm [45], [b]RMSDs were calculated from the backbone C^{α} atoms of the superimposed structures.

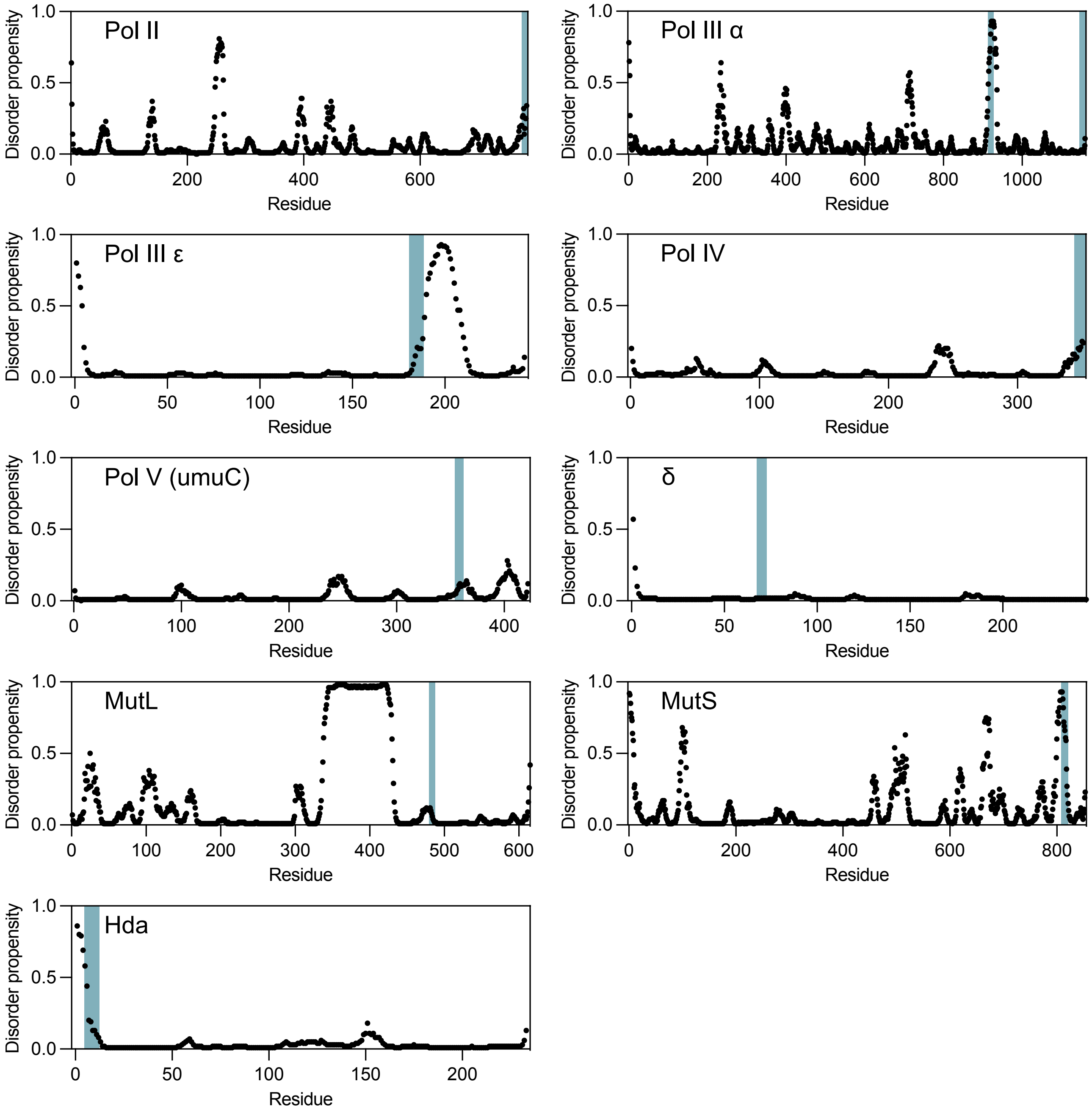

Fig. S1 Disorder prediction of selected β-clamp interaction partners. The disorder propensity is plotted for each residue and was predicted using DISOPRED3 (http://bioinf.cs.ucl.ac.uk/psipred) [137]. Blue boxes indicate the location of the β-clamp binding motifs (CBMs).

```
                      1         10        20        30        40        50            60             70        80
E. coli (1MMI)        --MKFTVEREHLLKPLQQVSGPLGGRPTLPILGNLLLQVADGTLSLTGTDLEMEMVARVAL-----V--QP-H-EPGATTVPARKFFDICRGLPEGAEIA 89
E. cloacae (6AMQ)     --MKFTVEREHLLKPLQQVSG-------LPILGNLLLQVADGTLSLTGTDLEMEMIARVTL-----S--QP-H-EAGATTVPARKFFDICRGLPEGAEIA 82
P. aeruginosa (6AMS)  --MHFTIQREALLKPLQLVAGVV---T-LPVLSNVLLVVEGQQLSLTGTDLEVELVGRVVL-----E--DA-A-EPGEITVPARKLMDICKSLPNDVLID 85
A. baumannii (6AP4)   -HMRLKIAKESLLNVLSHVVGAVERRHTLNILSNVKIQTNAQALTITGSDLEVELVASTAL-----S--EGACLEAGETTVPARKLMEICKSLPTAALID 92
M. tuberculosis (3P16) -DLTFRLLRESFADAVSWVAKNLPARPAVPVLSGVLLTGSDNGLTISGFDYEVSAEAQVGA--------EI-V-SPGSVLVSGRLLSDITRALPN-KPVD 88
B. subtilis (4TR6)    SHMKFTIQKDRLVESVQDVLKAVSSRTTIPILTGIKIVASDDGVSFTGSDSDISIESFIPKEEGDKEIVTI-E-QPGSIVLQARFFSEIVKKLP-MATVE 97
S. pyogenes (2AVT)    -MIQFSINRTLFIHALNTTKRAISTKNAIPILSSIKIEVTSTGVTLTGSNGQISIENTIPV----GL--LI-T-SPGAILLEASFFINIISSLP-DISIN 90
H. pylori (4S3I)      --MKISVSKNDLENALRYLQAFLDKKDASSIASHIHLEVIKEKLFLKASDSDIGLKSYIFT-----Q--SS-D-KEGVGTINGKKFLDIISCLKD-SNII 88

                      90         100       110       120        130       140          150       160         170       180
E. coli (1MMI)        VQLEG-E-RMLVRSGRSRFSLSTLPAADFPNLDD-WQSEVEFTLPQATMKRLIEATQFSMAH--QDVRYYLNGMLFETE--GEELRTVATDGHRLAVCSM 182
E. cloacae (6AMQ)     VQLEG-D-RMLVRSGRSRFSLSTLPAADFPNLDD-WQSEVEFTLPQATMKRLIEATQFSMAH--QDVRYYLNGMLFETE--GEELRTVATDGHRLAVCSM 175
P. aeruginosa (6AMS)  IRVEE-Q-KLLVKAGRSRFTLSTLPANDFPTVEE-GPGSLNFSIAQSKLRRLIDRTSFAMAQ--QDVRYYLNGMLLEVN--GGTLRSVATDGHRLAMCSL 178
A. baumannii (6AP4)   LQITEDQ-RCILKSGNSRFVLGTLPAEDYPLLTTENSQGTQVQVTQRELKRLFEKTAFAMAV--QDVRFYLTGTLLEID--ENQLRAVTTDGHRLALCEI 187
M. tuberculosis (3P16) VHVEG-N-RVALTCGNARFSLPTMPVEDYPTLPT-LPE-ETGLLPAELFAEAISQVAIAAGRDDTLPML--TGIRVEIL--GETVVLAATDRFRLAVREL 180
B. subtilis (4TR6)    IEVQNQY-LTIIRSGKAEFNLNGLDADEYPHLPQ-IEEHHAIQIPTDLLKNLIRQTVFAVST--SETRPILTGVNWKVE--QSELLCTATDSHRLALRKA 191
S. pyogenes (2AVT)    VKEIE-QHQVVLTSGKSEITLKGKDVDQYPRLQE-VSTENPLILKTKLLKSIIAETAFAASL--QESRPILTGVHIVLSN-HKDFKAVATDSHRMSQRLI 185
H. pylori (4S3I)      LETKD-D-SLAIKQNKSSFKLPMFDADEFPEFPV-IDPKVSIEVNAPFLVDAFKKIAPVIEQ--TSHKRELAGILMQFDQKHQTLSVVGTDTKRLSYTQL 183

                                 190       200       210                220                 230       240       250       260
E. coli (1MMI)        PIGQ--S-L---P-SHSVIVPRKGVIELMRMLDGGDNPLRVQI-----GS--NNIRAHVG-------DFIFTSKLVDGRFPDYRRVLPKNPDKHLEAGCD 261
E. cloacae (6AMQ)     PIGD--S-L---P-NHSVIVPRKGVIELMRMLDGGDTPLRVQI-----GS--NNIRAHVG-------DFVFTSKLVDGRFPDYRRVLPKNPDKTLEAGCD 254
P. aeruginosa (6AMS)  DAQI--P-S---QDRHQVIVPRKGILELARLLTEQDGEVGIVL-----GQ--HHIRATTG-------EFTFTSKLVDGKFPDYERVLPRGGDKLVVGDRQ 258
A. baumannii (6AP4)   LASS--TSS---Q-LVQAIVPRKAVGELQRLLSIEDEQLTLLI-----GR--ELLNVTINTPEQGDITVRFTTKLIDGKFPDYRRVIPRGGDKHVLIGHD 274
M. tuberculosis (3P16) KWSASSP-D---I-EAAVLVPAKTLAEAAKAGI-GGSDVRLSLGTGPGVGKDGLLGISGN-------GKRSTTRLLDAEFPKFRQLLPTEHTAVATMDVA 267
B. subtilis (4TR6)    KLDI--P-ED--R-SYNVVIPGKSLTELSKILDDNQELVDIVI-----TE--TQVLFKAK-------NVLFFSRLLDGNYPDTTSLIPQDSKTEIIVNTK 271
S. pyogenes (2AVT)    TLDN--T-S---A-DFMVVLPSKSLREFSAVFTDDIETVEVFF-----SP--SQILFRSE-------HISFYTRLLEGNYPDTDRLLMTEFETEVVFNTQ 264
H. pylori (4S3I)      EKIS--I-HSTEE-DISCILPKRALLEILKLF-YE--NFSFKS-----DG--MLAVIENE-------MHTFFTKLIDGNYPDYQKILPKEYISSFTLGKE 262

                             270        280       290       300       310       320       330       340       350
E. coli (1MMI)        LLKQAFARAAILSN-E-KFRGVRLYVS-ENQLKITAN-NPEQEEAEEILDV-T-YSGA-EMEIGFNVSYVLDVLNALKCENVRMMLTDSVSSVQIEDAAS 354
E. cloacae (6AMQ)     SLKQAFARAAILSN-E-KFRGVRLYVS-ENQIKITAN-NPEQEEAEEILDV-T-YAGT-EMEIGFNVSYVLDVLNALKCENVRILLTDSVSSVQIEDAAS 347
P. aeruginosa (6AMS)  QLREAFSRTAILSN-E-KYRGIRLQLS-NGLLKIQAN-NPEQEEAEEEVQV-E-YNGG-NLEIGFNVSYLLDVLGVIGTEQVRFILSDSNSSALVHEADN 351
A. baumannii (6AP4)   VFKQSLQRVAILSN-E-KLRGVFLNFN-QDSLQLRAN-NPEQDEAIEDLAI-Q-YQSA-PLEMSFNAQYLLDVLGVLDGDDVNMSMTEANQSVLVQDPAH 367
M. tuberculosis (3P16) ELIEAIKLVALVAD-R--GAQVRMEFA-DGSVRLSAG-ADDVGRAEEDLVV-D-YAGE-PLTIAFNPTYLTDGLSSLRSERVSFGFTTAGKPALLRPVSG 359
B. subtilis (4TR6)    EFLQAIDRASLLAR-EGRNNVVKLSAKPAESIEISSN-SPEIGKVVEAIVADQ-IEGE-ELNISFSPKYMLDALKVLEGAEIRVSFTGAMRPFLIRTPND 367
S. pyogenes (2AVT)    SLRHAMERAFLISNAT-QNGTVKLEIT-QNHISAHVN-SPEVGKVNEDLDIVS-QSGS-DLTISFNPTYLIESLKAIKSETVKIHFLSPVRPFTLTPGDE 359
H. pylori (4S3I)      EFKESIKLCSSLS------STIKLTLE-KNNALFESLDSEHSETAKTSVEIEKGLDIEKAFHLGVNAKFFLEALNALGTTQFVLRCNEPSSPFLIQESLD 355

                                360
E. coli (1MMI)        ----Q-------SAAYVVMPMRL 366
E. cloacae (6AMQ)     ----Q-------SAAYVVMPMRL 359
P. aeruginosa (6AMS)  ----D-------DSAYVVMPMRL 363
A. baumannii (6AP4)   ----P-------DQTYVVMPMR- 378
M. tuberculosis (3P16) DAVST-------DYVYLLMPVRL 375
B. subtilis (4TR6)    ----E-------TIVQLILPVRT 379
S. pyogenes (2AVT)    ---EE-------SFIQLITPVRT 372
H. pylori (4S3I)      ----EKQSHLNAKISTLMMPITL 374
```

Fig. S2 Structural sequence alignment of β-clamps from different organisms. Sequence alignments are based on the structure alignment using the TM-align algorithm. Note that sequences not visible in the PDB structure are not present in this alignment. Sequence conservations are visualized using the Color Align Conservation tool at https://www.bioinformatics.org with a percentage identity or similarity cut-off of 70%. Amino acids are grouped according to their characteristics; positive = H, R ,K; negative = D, E; Neutral = S, T, N, Q; Aliphatic = A, V, L, I, M; Aromatic = F, Y, W; Pro & Gly = P, G; Cys = C. Numbers on top of the sequences are amino acid positions of the E. coli β-clamp sequence

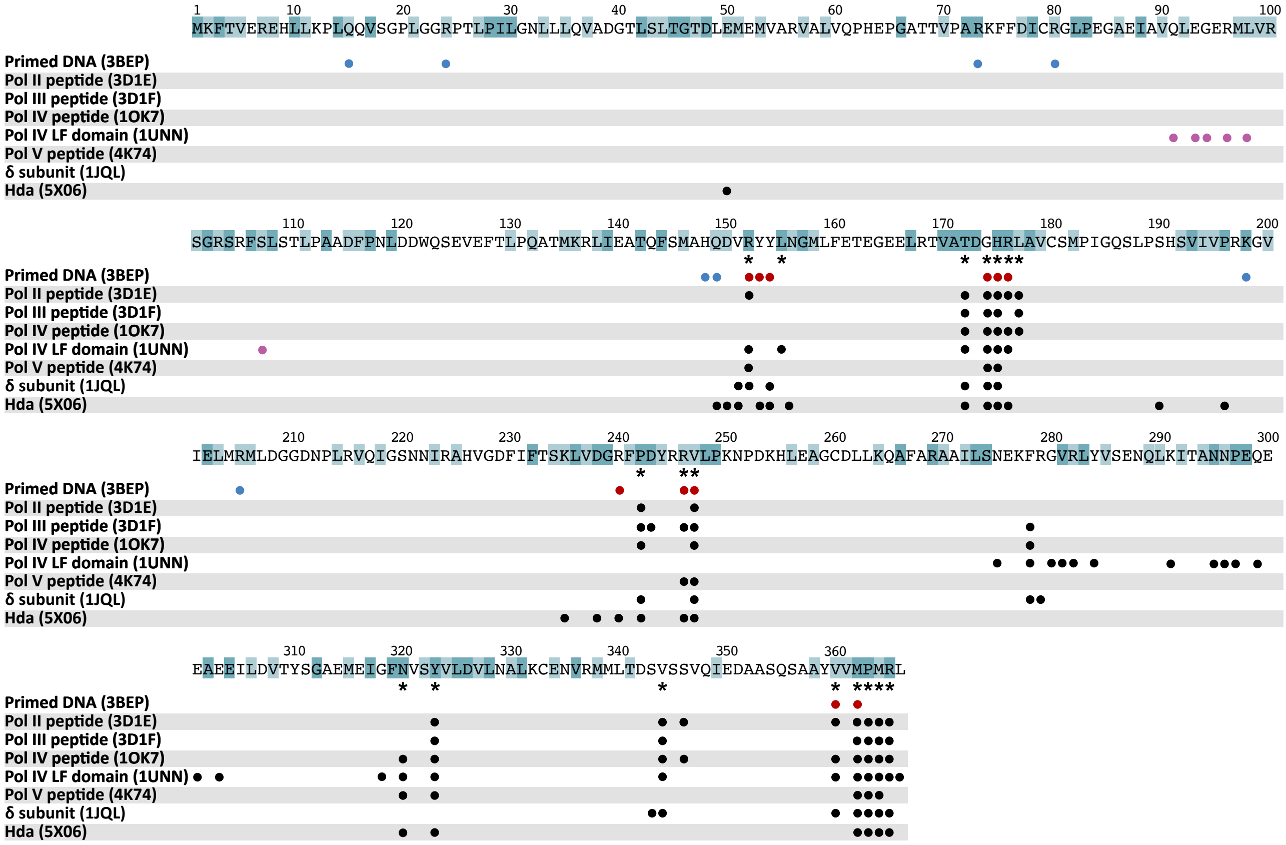

Fig. S3 Overview of β-clamp interacting residues and their conservation. PDBsum [138] was used to identify β-clamp residues that are in close proximity (≤ 3.9 Å) to the protein or peptide interaction partners (PDB ID in the parenthesis) in the crystal structures (black dots). The interacting residues between β-clamp and primed DNA are based on the interactions reported in the paper by Georgescu et al. [5]. β-clamp residues are coloured according to their structural sequence conservation as shown in Fig. S2. Stars indicates residues that constitute the canonical CBM binding pocket. Blue dots of the primed DNA interaction indicate interactions with the inner hole of β-clamp and red dots indicate interactions to residues of an adjacent β-clamp molecule in the crystal lattice. Purple dots of the Pol IV LF domain interaction indicate interactions formed by the other subunit of the β-clamp dimer.
